# Equilibrium shape and surface termination of supported magnetite nanoparticles

**DOI:** 10.1038/s42004-026-02008-4

**Published:** 2026-04-11

**Authors:** Mohammad Ebrahim Haji Naghi Tehrani, Daniel Silvan Dolling, Jan-Christian Schober, Esko Erick Beck, Mona Kohantorabi, Arno Jeromin, Ludwig J. V. Ahrens-Iwers, Thomas F. Keller, Vedran Vonk, Gregor B. Vonbun-Feldbauer, Heshmat Noei, Andreas Stierle

**Affiliations:** 1 Centre for X-ray and Nano Science CXNS, Deutsches Elektronen-Synchrotron DESY, Hamburg, Germany; 2 Physics Department, University of Hamburg, Hamburg, Germany; 3 Institute for Interface Physics and Engineering, Hamburg University of Technology, Hamburg, Germany; 4 Institute of Surface Science, Helmholtz-Zentrum Hereon, Geesthacht, Germany

**Keywords:** Nanoparticles, Surface spectroscopy

## Abstract

The equilibrium shape and surface termination of magnetite nanoparticles (NPs) are fundamental properties that determine the physical and chemical properties of the supercrystal structure of oleic acid-coated magnetite NPs. Here, we studied the equilibrium shape of magnetite (Fe_3_O_4_) NPs supported by Al_2_O_3_(0001) single-crystalline surfaces. We report the growth of epitaxial (111)-oriented NPs exhibiting a triangular shape with a height-to-diameter aspect ratio of 0.42 over a wide growth temperature range. We probed the surface termination of the NP facets by adsorbing formic acid as a prototypical molecule representing the adsorption behavior of oleic acid. We identified infrared absorption bands characteristic of dissociative adsorption on (111) facets with the iron tetrahedral (Fe-tet_1_) termination, as well as on mixed-terminated (100) side facets. Our experimental findings are supported by predictions of the NP shape using surface-free energy calculations from ab initio thermodynamics. The experimentally observed nanoparticle shape can only be rationalized by the presence of bulk-terminated {100} type facets. Such a fundamental understanding of the shape and surface terminations of oxide nanoparticles is crucial for tailoring the properties of hybrid hierarchical materials and drug carriers and for their development.

## Abstract (web summary)

The equilibrium shape and surface termination of nanoparticles (NPs) determine the interfacial binding mechanisms at play during the synthesis of NP-based materials. Here, the authors combine experiments and ab initio thermodynamics and demonstrate that (111)-oriented magnetite NPs supported by Al_2_O_3_(0001) single-crystalline surfaces are bulk-truncated by Fe-terminated {111} and mixed-terminated {100} facets.

## Introduction

Hierarchical nanostructures are designed by assembling nanoscale building blocks into ordered superstructures^1,2^. Magnetite nanoparticles play an important role as building blocks in hierarchical materials with exceptional mechanical and structural properties due to their unique physical and chemical properties^3–8^. This new class of materials can be fabricated by adsorbing and crosslinking of fatty acids, such as oleic acid, as an interphase between the magnetite NPs^7,9–11^. These materials exhibit outstanding bending modulus along with high hardness and strength^7,12^. Important parameters governing these properties are the shape and size of the nanoparticles^7,12^, which can be controlled with high precision during synthesis^13^. Furthermore, the adsorption strength of the organic molecules on the nanoparticle facets is decisive to the supercrystal’s mechanical properties. It was shown that the packing density of oleic acid is higher on the (111) facet compared to the (001) facet, resulting in stronger crosslinking^14^. However, the surface terminations of NP facets, which determine the interfacial binding mechanisms of organic molecules, such as oleic acid, have not been comprehensively investigated.

**Fig. 1:**
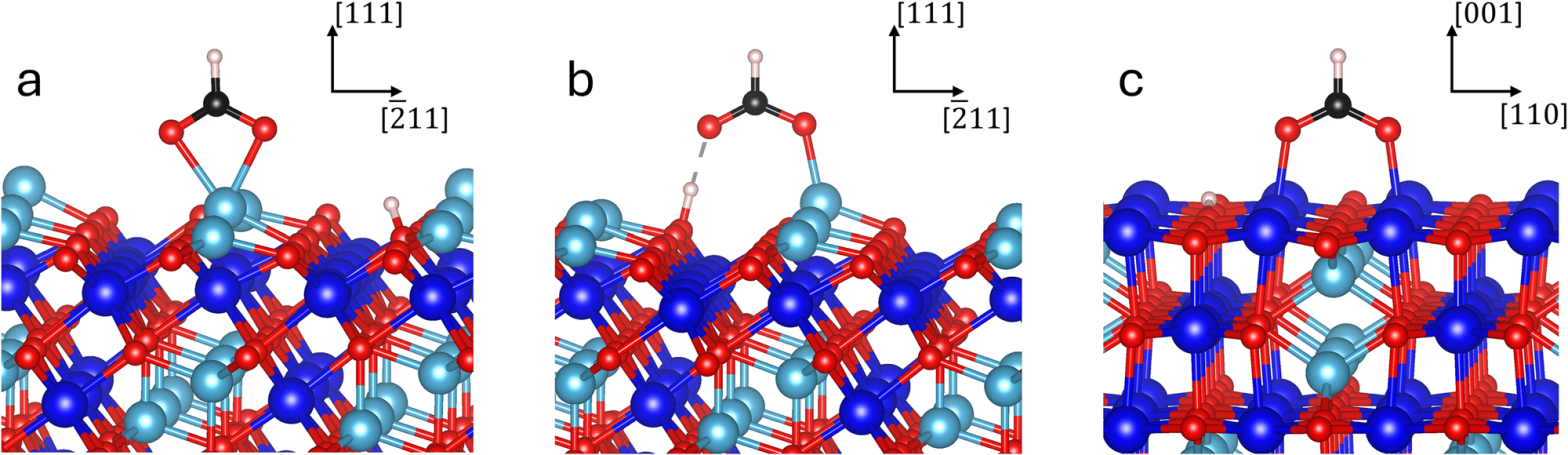
Adsorption structures of formate. (**a**) chelating, (**b**) quasi-bidentate and (**c**) bridging bidentate formate on the magnetite single crystalline surfaces, namely (111) in (**a**, **b**), (001) in (**c**). Fe_tet_, Fe_oct_ and oxygen ions are shown with light blue, dark blue, and red, respectively.

From a thermodynamic point of view, (001) and (111) are the most stable magnetite surfaces for a wide range of temperatures and oxygen pressures, including the experimental conditions used in this study^13^. This suggests that they may dominate the Wulff shape of magnetite nanoparticles under equilibrium conditions. The termination of these stable surfaces depends strongly on the experimental conditions^15–18^. For typical surface science conditions (oxygen partial pressure of 10^-12^–10^-6 ^mbar) and temperatures (T = 300 – 900 K), the following terminations show the highest stability^18–21^. The (001) surface is terminated by a mixed oxygen and iron layer in truncated octahedral sites. Under the above-mentioned conditions, this surface often shows a subsurface cation vacancy (SCV) reconstruction with additional interstitial tetrahedral Fe in the surface layer and ordered subsurface octahedral Fe vacancies^19,22^. Upon adsorption, e.g., formic acid, this reconstruction is lifted, and a distorted bulk truncated (DBT) termination is obtained without interstitial Fe and subsurface Fe vacancies^23–25^. For the (111) surface, the Fe-tet_1_ termination exhibits Fe ions on tetrahedral sites above a closed-packed oxygen layer under UHV conditions^20,21,26^. Adsorption of formic acid was studied previously on magnetite (001) and (111) single-crystalline surfaces. Formic acid represents the adsorption behavior of oleic acid, since it binds to the magnetite surface with the same carboxylic functional group^14^. It was shown that formic acid adsorbs dissociatively on these surfaces through the following main adsorption geometries^23,24,26–28^: adsorption of formate in quasi-bidentate and chelating geometries was evidenced on the magnetite (111) surface^14,26^ (see Fig. 1a, b). On the (001) surface, however, formate species were reported to adsorb in the bridging bidentate geometry^14,23,24^ (see Fig. 1c), lifting the subsurface cation vacancy reconstruction over a wide range of oxygen chemical potential^19^.

The pertinent literature lacks a connection between the studies on the magnetite single-crystalline surfaces and the magnetite NP systems. The fundamental understanding of the physico-chemical properties of magnetite NPs and their interaction with organic molecules has not been explored comprehensively in the literature. The contribution of different NP facets and the possible shape alterations after the adsorption of organic molecules are not well understood. In addition, the surface termination of the NP facets, which controls the adsorption behavior on a microscopic level, has not been thoroughly investigated. On a more fundamental level, the thermodynamic equilibrium (Wulff) shape of oxide nanoparticles is experimentally not well established. Compared to pure metals, not only the facet orientation dependent Gibbs free energy determines the shape, but also different possible facet surface terminations.

Here, we investigated the atomic structure and shape of magnetite NPs after growth in the temperature range from 423 K to 773 K by reactive physical vapor deposition on Al_2_O_3_(0001). Al_2_O_3_ single crystals were used due to their chemical inertness at high temperatures and negligible interaction with formic acid at room temperature. Scanning electron microscopy (SEM), X-ray reflectivity (XRR), grazing incidence X-ray diffraction (GIXRD) and X-ray photoelectron spectroscopy (XPS) were applied to determine the morphology, structure and phase of the NPs. We have probed the NPs’ surface termination and facets using Fourier Transform Infrared Reflection-Absorption Spectroscopy (FT-IRRAS) by employing formic acid. Formic acid is the simplest carboxylic acid, and its adsorption behavior can be extrapolated to understand the adsorption of larger carboxylic acids such as oleic acid^14,26^. To support our experimental findings, we predict the equilibrium shape of the nanoparticles by Wulff and Winterbottom constructions based on Gibbs free energies obtained from ab initio thermodynamics for different nanoparticle facet surface terminations.

## Results and discussion

In the first part of the results section, we will present the structural and morphological properties of the magnetite nanoparticles. This is followed by the FT-IRRAS formic acid adsorption studies in the second part and theoretical investigations in the third part of this section. The nanoparticles were grown by reactive physical vapor deposition on precleaned Al_2_O_3_ (0001) single crystal substrates at varying substrate temperatures (see Methods section for details). In brief, iron was evaporated from a rod by e-beam bombardment in an oxygen background pressure of 8·10^−^^7^ mbar. Nominally around 3 nm Fe_3_O_4_ was deposited for all samples.

### Morphological and structural characterization

**Fig. 2:**
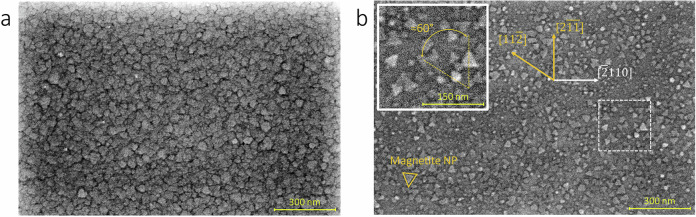
Room temperature SEM images of magnetite NPs. (**a**) after growth at 573 K and (**b**) after growth at 773 K both on Al_2_O_3_(0001) substrates. The two orientations of the NPs are indicated by dashed lines in the inset. The crystallographic orientation of the NPs with respect to the substrate is shown in orange and white, respectively.

The morphology of the NPs was investigated by SEM at room temperature. The morphology of the NPs grown at 423 K could not be imaged due to a strong charging effect on the sample (Figure S1). However, the microscopy images of the NPs grown at 573 K show NPs with irregular and for some cases triangular shapes (see Fig. 2a). Furthermore, the growth of more triangular-shaped and faceted NPs was observed at an increased growth temperature to 773 K, see Fig. 2b. The size distribution of the NPs grown at 773 K was investigated using an image-based perimeter distribution analysis (see Figure S2 and SI). The distribution curve exhibits a maximum at ≈6.9 nm, a mean value of 39 nm and a range spanning from 4 to 250 nm. Additionally, the size distribution exhibits a long tail towards larger diameters, indicating heterogeneous nucleation and growth (see Figure S2)^29^. Furthermore, Fig. 2b indicates two preferred orientations of the NPs rotated by 60° relative to each other. This suggests epitaxial growth of the NPs on Al_2_O_3_(0001) in two different domains, as shown in the inset of Fig. 2b.

**Fig. 3:**
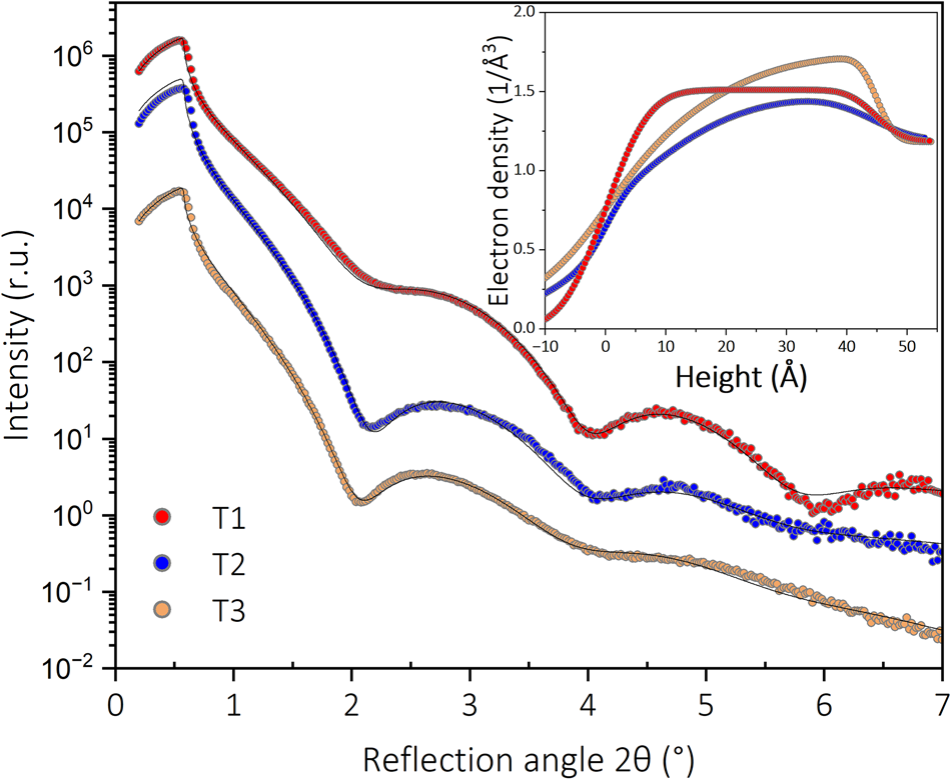
X-ray reflectivity curves and fitted electron density profiles of the magnetite NPs grown at different temperatures. T1: 423 K, T2: 573 K, T3: 773 K, dots: measured data, black lines: fit curves. The XRR curves are shifted on the y-axis for clarity.

XRR was carried out to determine the morphology of the nanoparticles (NPs). From XRR, the total electron density profile perpendicular to the sample surface can be determined, disclosing information about the average NP height, surface coverage and height distribution^30^. Figure 3 illustrates the evolution of the reflectivity curves for different growth temperatures. At first glance, the XRR curves exhibit minima at similar angular positions, indicating a consistent height of the magnetite NPs for all growth temperatures. Furthermore, increasing the growth temperature from 423 K to 773 K results in a damping of the oscillation’s amplitude, reflecting an increase in the NP height distribution. For the quantitative analysis, one layer of magnetite with variables of thickness, electron density, surface and interfacial roughness on the alumina support was used^30,31^. A second ultrathin layer was included in the model for the samples grown at 573 K and 773 K to improve the fit quality. The fit based on the introduced model describes the measurements well (see Fig. 3 and Table S1 for the fit parameters). The estimated thickness is around 4.2 nm for all growth temperatures, which reflects the average height of the NPs. Additionally, the coverage of the NPs varies between 72 and 83% across all the growth temperatures, in accordance with the high coverage observed in the SEM images. Furthermore, by increasing the growth temperature from 423 K to 773 K, the roughness of the magnetite NP layer increases from 6 to 17 Å, which can be explained by an increase in NP height distribution.

**Fig. 4:**
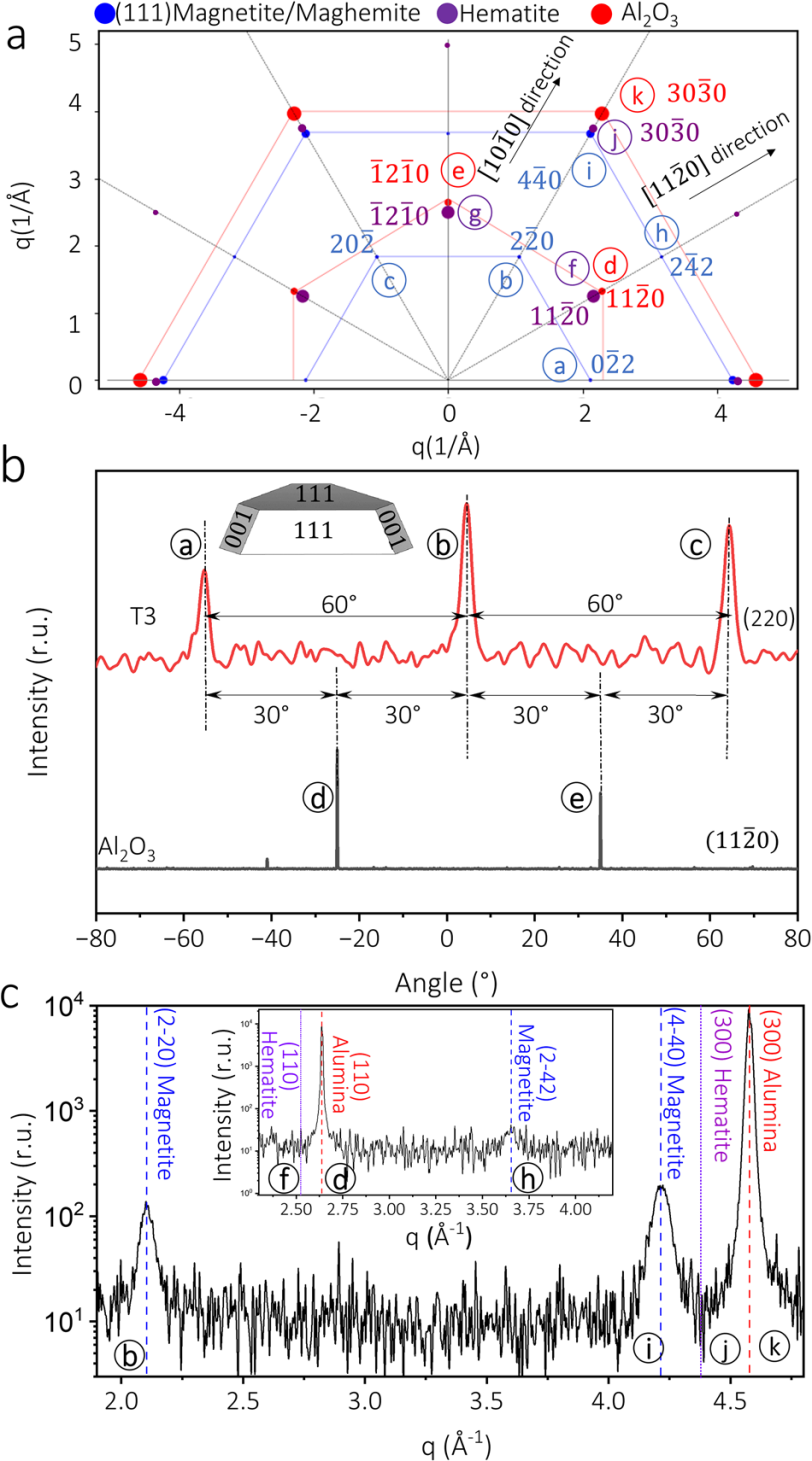
GIXRD characterization of the nanoparticles. **a** In-plane reciprocal map of alumina (0001), (111)-oriented magnetite and hematite (0001); the diameter of the points in the map corresponds to the intensity of the Bragg peak^69^. **b** In-plane rocking scan of the magnetite NPs grown at 773 K (red line) compared to the substrate (black line), and (**c**) In-plane radial scan in the alumina $(10\bar{1}0)$ direction, inset: in-plane radial scan in the alumina $(11\bar{2}0)$ direction.

The crystalline structure and epitaxy of the NPs were explored by recording grazing incidence in-plane diffraction patterns. The possibility of the formation of (111), (001) and (110) oriented particles was investigated by recording the in-plane diffraction signals from the (220), (222) and (400) magnetite Bragg reflections (see Figure S3). No peaks in the in-plane rocking scans through (222) and (400) reflections were detected. Therefore, the possibility of (100) and (110) oriented magnetite NPs can be excluded. On the other hand, in the in-plane rocking scan through (220) reflection of magnetite, 60° repeated peaks were recorded for all the growth temperatures (Fig. 4b and Figure S3). These reflections correspond to reflections a - c in the in-plane reciprocal map of (111)-oriented magnetite (Fig. 4a). Furthermore, the FWHM of the reflections decreases with increasing growth temperature (Figure S3d). The $(11\bar{2}0)$ reflection of Al_2_O_3_(0001) substrate was adopted as a reference Bragg peak to determine the orientation of the NPs with respect to the substrate (see Fig. 4b). According to the in-plane rocking scans (Fig. 4b), the (220) magnetite reflection is 30° rotated with respect to the $(11\bar{2}0)$ reflection of alumina. The result is in line with an in-plane alignment of the Al_2_O_3_ $(11\bar{2}0)$ planes with the Fe_3_O_4_ $(1\bar{2}1)$ planes (Fig. 4a). We can conclude that the NPs adopt a preferred orientation with respect to the substrate in the whole growth temperature range. The formation of (111)-oriented NPs is induced by the hexagonal surface structure of the Al_2_O_3_(0001) substrate^32^. Taking into account the two possible in-plane orientations of the nanoparticles observed in the SEM images, we can conclude that the two types of domains correspond to NPs with either ABC or CBA stacking of the closed-packed (111) planes. A similar growth orientation was earlier reported for the growth of Pt - Rh NPs on the alumina substrate^32,33^.

In-plane radial scans along the alumina $(10\bar{1}0)$ and $(11\bar{2}0)$ directions were carried out to further explore the crystal structure and orientation of the NPs (see Fig. 4c). The in-plane radial scans exhibit the corresponding reflections for (111)-oriented magnetite NPs. In addition, no reflections corresponding to the hematite phase were detected in the diffraction patterns (Fig. 4c). The in-plane lattice parameter of the NPs was determined from the position of the $\left(2\bar{2}0\right)$ peak in the in-plane radial scans in order to investigate the possibilities of epitaxial strain and maghemite formation (see Figure S4 and Table S2). The Bragg angles of the $\left(2\bar{2}0\right)$ and $\left(4\bar{4}0\right)$ reflections of the NPs shifted to lower angles compared to the bulk magnetite values, indicating larger in-plane lattice parameters (Fig. 4c and Table S2). Hence, the possibility of in-plane epitaxial strain between the NPs and the alumina substrate can be excluded since a peak shift to higher Bragg angles would be expected. Additionally, the in-plane lattice parameter of the NPs is notably higher than the bulk lattice parameter of maghemite. Maghemite (γ-Fe_2_O_3_) possesses an inverse spinel structure similar to the magnetite crystal structure with a lattice parameter of 8.351 Å, which is smaller than that of magnetite^34^. Magnetite, therefore, is the dominant phase of the NPs, and the larger in-plane lattice parameter of the NPs can be attributed to oxygen vacancies in the NP structure^35,36^.

The average diameter and height of the NPs were further investigated by analyzing the Bragg peak width in *h* and *l* directions (see Figure S5 and Table S3)^37^. The $\left(3\bar{1}1\right)$ Bragg peak of magnetite was chosen since this type of reflection is the most intense. The average height of 4.2 nm determined from the XRR data is in good agreement with the average height of 4.4 nm determined from the line scans (see Table S3), which infers a fully crystalline NP structure. Furthermore, the average diameter of the NPs is 10 nm, agreeing well with the maximum of the size distribution from the SEM image analysis. According to Table S3, the average height to diameter aspect ratio of the NPs is approximately 0.42. Furthermore, it remained constant for different growth temperatures, and we take it, therefore, as an indication that the NPs exhibit an equilibrium shape which is independent of the growth temperature. Based on the height to diameter aspect ratio of the NPs and the SEM results, we propose the preliminary model shown in the inset of Fig. 4b. The model depicts a triangular-shaped (111)-oriented NP, featuring top (111) and side (001)/(111) facets, where one type of facet is more dominant than the other. From the present data, however, we cannot determine which type of side facet is more dominant.

### Spectroscopic characterization by XPS and FT-IRRAS

To further investigate the oxidation state of the NPs, XPS analysis was carried out directly after growth under UHV conditions without exposing the samples to air. The Fe 2p core level spectrum exhibits two peaks located at 710.9 and 724.5 eV in Figure S6. The spin - orbit split of Fe 2p_3/2_ and Fe 2p_1/2_ peaks is identical to the one recorded on a clean and adsorbate - free magnetite single crystalline sample^19^ (see Figure S6). The XPS analysis of the same sample after exposure to air for 99 days shows two Fe_sat_^3+^ satellite shoulders at 719.1 and 733.2 eV. This suggests a possibility of the partial phase transition from magnetite (Fe_3_O_4_) to maghemite (γ-Fe_2_O_3_) at the surface of the NPs via oxidation in air^38^.

Furthermore, we studied the adsorption of formic acid on magnetite NPs using polarization-dependent FT - IRRAS to probe possible adsorption geometries and obtain information about the surface termination and orientation of the facets. The *p*-polarized light can be decomposed into *p*_*t*_ (tangential) and *p*_*n*_ (normal) components, which are parallel and perpendicular to the sample surface, respectively (see Figure S7). Consequently, the *p*_*t*_ and *p*_*n*_ components excite parallel and perpendicular dynamic dipole moments with respect to the sample surface, respectively. Similar to the *p*_*t*_ component, the *s-*polarized light excites dynamic dipole moments parallel to the sample surface (perpendicular to the plane of incidence)^39,40^.

The reflectance for *p*_*n*_ and *s*-polarized light possesses the same signs, and they exhibit opposite signs relative to the *p*_*t*_ component^40^. The sign of the FT-IRRAS reflectance signal for supported NPs is determined by the dielectric properties of the substrate^39^. Our system consists of magnetite NPs and a single crystal of alumina as a substrate. Previously, we observed positive *p*_*n*_ and *s* reflectance signals for PtPd nanoparticles on Al_2_O_3_ (0001), while the *p*_*t*_ component exhibits a negative signal^32^. Additionally, parallel dynamic dipole moments on an oxide surface can be probed contrary to metallic surfaces, where the excitation of parallel dynamic dipole moments, due to the surface selection rule, cannot be probed^41^.

In Figure S8 and Table 1, we give an overview of possible excitations. On the top facet of the NPs, the symmetric vibrations (*ν*_*s*_) of the O-C-O stretching mode can give rise to a dynamic dipole moment perpendicular to the surface, which is excited by the *p*_*n*_ component (see Figure S8a and Table 1). In addition, the dipole moment of the asymmetric O-C-O stretching vibration (*ν*_*as*_) on the top facet is parallel to the surface (see Figure S8b, c) and the corresponding dipole is excited by *p*_*t*_ *and s-*polarized components. To explain the excitation of the symmetric and asymmetric O-C-O stretching vibrations on the side facets, the dipole moments need to be decomposed into their perpendicular and parallel components with respect to the sample surface (see Table 1). The decomposed component parallel to the sample surface is excited by *p*_*t*_ and *s* polarized light, while the decomposed component perpendicular to the sample surface is excited by *p*_*n*_. In IR spectroscopy, the intensity of the signals (I) is proportional to the scalar product of the dynamic dipole moment $(\vec{d})$ and the electric field $(\vec{E})$. This relation can be described further by the following formula:

1 $$I\propto \vec{d}.\,\vec{E}=|{{\rm{d}}}|.{|}{{\rm{E}}}|.\cos {{\rm{\alpha }}}$$

where α is the angle between the dynamic dipole moment and the electric field^39^. The angle between the top and side (111) facets is ~70.5°^42^. As a result, the symmetric dynamic dipole moments on the side facets are ~70.5° rotated with respect to the substrate surface normal. Due to this fact, on the side facets, pn mainly excites *ν*_*s*_ (with a positive sign) and *ν*_*s*_ as well as *ν*_*as*_ are excited by *p*_*t*_ (both with a negative sign) (see Figure S8 and Table 1). Consequently, there is a compensation effect (due to the opposite signs of *p*_*t*_ and *p*_*n*_^40^) between these components for the top and the side facet signals, which leads to a lower intensity of the symmetric bands compared to that of the asymmetric bands in the *p*-polarized spectrum (see Table 1).

**Table 1:** Excited dynamic dipoles on the top and side (111) facets of magnetite nanoparticles under *s* and *p* polarized light and their corresponding signs

Polarized beam		Excited dipole	Facet	sign
*s*		*D*_*as*_	Top	+
		≃*D*_*s*_sin(70.5°)	Side	+
*p*	*p*_*t*_	*D*_*as*_	Top	−
		≃*D*_*s*_sin(70.5°)	Side	−
	*p*_*n*_	*D*_*s*_	Top	+
		≃*D*_*as*_sin(70.5°)	Side	+

**Fig. 5:**
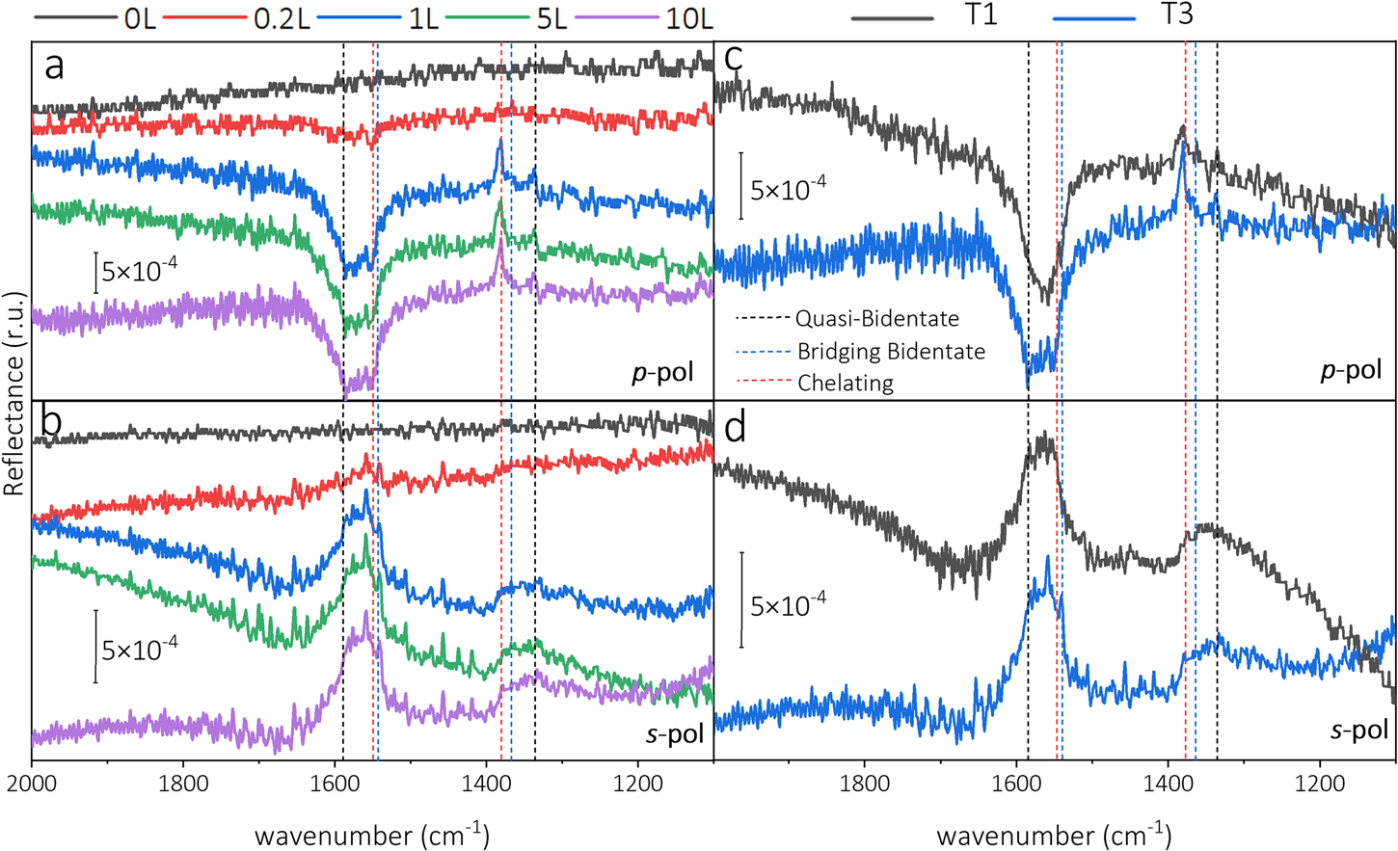
IRRAS of formic acid adsorbed on magnetite nanoparticles. IRRAS spectra of magnetite NPs grown at 773 K recorded after dosing to 0.2, 1, 5, and 10 L of formic acid at room temperature measured with (**a**) *p* and (**b**) *s*-polarized light. IRRAS spectra of magnetite NPs grown at 423 K after dosing 10 L of formic acid compared to those of magnetite NPs grown at 773 K measured with (**c**) *p* and (**d**) *s*-polarized light. The position of the bridging bidentate, chelating and quasi-bidentate vibrational bands reported for magnetite (001) and (111) single crystalline surfaces are shown by blue, red and black short dash lines, respectively.

Figure 5 shows the experimental results for the samples grown at 423 K and 773 K. As the most predominant features, the *p*-polarized spectra exhibit a broad negative band in the region of the asymmetric O-C-O stretching bands of formate (1500 - 1650 cm^−^^1^) originating from the excited adsorbates by the *p*_*t*_ component of the light^26^. The width of the band suggests different asymmetric vibrations, which are mostly from two individual bands located at 1549 and 1587 cm^−1^. In addition, there are two positive bands excited by the *p*_*n*_ component of the light at 1336 and 1381 cm^−1^. Fig. 5 also depicts broad positive bands in the *s*-polarized spectra in the regions of 1500 – 1650 cm^−1^ and 1330 – 1380 cm^−1^, both exhibiting the same sign as the *p*_*n*_ component (see Table 1). The positions of the bands in the *s* and *p*-polarized spectra match with the reported symmetric and asymmetric vibrations of dissociatively adsorbed formic acid on the magnetite (111) single crystalline surface with Fe-tet_1_ termination (see Table 2)^26^.

**Table 2:** Overview of vibrational bands of dissociative adsorption of formic acid on magnetite NPs compared to the magnetite (001) and (111) single crystalline surfaces

	Bridging bidentate		Quasi-bidentate		Chelating	
	Symmetric (cm^−^^1^)	Asymmetric (cm^−1^)	Symmetric (cm^−1^)	Asymmetric (cm^−1^)	Symmetric (cm^−1^)	Asymmetric (cm^−1^)
(001)	1368	1544	—	—	—	—
(111)	—	—	1338	1588	1380	1548
NPs	≈ 1368	—	1336	≈ 1587	1381	≈ 1549

The Fano line shape bands, observed for magnetite single crystals, are absent in the NP spectra. One possible reason for this is that the optical properties of the Al_2_O_3_ substrate dominate the reflectance, similar to the case of Fe_3_O_4_ film on Pt(111), where no Fano-type line was observed^43^. The intensity of the asymmetric and symmetric bands with positive signs in the *s*-polarized spectra originates from formate on the top and side facets of the NPs, respectively (see Figure S8c, d and Table 1). The shape of the symmetric O-C-O stretching bands in the *s*-polarized spectra is dissimilar to that of the *p*-polarized spectra. We assume that the minor contribution of the symmetric stretching band of bridging bidentate formate, which typically adsorbs on the (001) surface at 1368 cm^−1^, gives rise to a broad band in the region of 1330 – 1380 cm^−1^ ^23,24^. This can be taken as an indication of relatively small (001) side facets compared to the (111) facets. Similar to the excitation on the (111) side facets, the symmetric vibration of the bridging bidentate formates on the (001) side facets (with a positive signal) can be probed by the *s* component of the light (see Figure S8d). Nevertheless, the NP is dominated by (111) facets, and the (001) side facets make a minor contribution, see Figure S8. The size and shape distribution of the NPs and adsorption on higher index facets at the edges of the nanoparticles may contribute to the IR data. However, it is challenging to pinpoint the exact role of individual parameters. For the nanoparticles grown at 423 K (Fig. 5b), the distinct vibration bands on the broader maxima (1330–1380 cm^−1^) are less well pronounced, which may indicate the presence of more higher index facets. Therefore, we can conclude that formic acid dissociates and dominantly binds to the (111)-oriented NPs in the quasi-bidentate and chelating adsorption geometries, very similar to the (111) surface (see Fig. 1)^26^. Since no adsorption band is visible at 1730 cm^−1^, both in *s* and *p* polarization, molecular adsorption on the {111} type facets can be excluded. Furthermore, the observed bands and their frequencies are in line with Fe-tet_1_ terminated (111) facets similar to the magnetite (111) single crystalline surface^26^. The amplitude of the bridging bidentate signal from {100} type facets is weaker than that of the corresponding species on the {111} type facets. This suggests a domination of (111) side facets, which can be explained by a higher formation energy of the (100) facet compared to that of the (111) facet (see the section on shape prediction). Additionally, upon dissociation of formic acid, the dissociated hydrogen atoms form hydroxyl groups with the surface oxygen of magnetite^26^.

The evolution of the s and *p*-polarized spectra (Fig. 5 and Figure S9) at different dosing steps of formic acid indicates that at low coverage of formic acid (0.2 L), formate mainly adsorbs in the chelating geometry, which gives rise to the symmetric and asymmetric stretching O-C-O bands at 1381 and 1549 cm^−1^, respectively. At higher coverage of formic acid, formate adsorption takes place in the chelating, quasi-bidentate and bidentate geometries.

### Nanoparticle shape prediction

To be able to further interpret the experimental results, we predicted the equilibrium shape of the NPs from the surface free energies for the most stable terminations of the (111) and (100) surfaces reported in the literature^18–21^. The surface free energies at finite temperatures were obtained from DFT calculations performing ab initio thermodynamics based on the approach of Reuter and Scheffler^44^. The surface energies from our DFT calculations at 0 K and the surface free energies from ab initio thermodynamics at the NP growth conditions are shown in Table 3 (temperatures from 450 to 800 K and 8·10^−7^ mbar oxygen pressure). The effective surface free energies $({\gamma }^{* })$ between the NP and the substrate were not obtained from DFT calculations of the interface, which would be computationally very demanding, but from the calculated surface free energies for (001) and (111) and the experimentally well accessible aspect ratios of the supported NPs using Eq. 3 (see Methods section). These (effective) surface free energies (see Table 3) were used to predict the shape of unsupported and supported NPs using the Wulff construction and Winterbottom construction, respectively, as implemented in the WulffPack code^45^. We have extended this code during this study to allow for negative effective surface energies, which are indispensable for the studied system (see Methods section). Considering the experimental growth conditions in this work (oxygen chemical potential in the range of −1.6 to −0.8 eV when assuming ideal gas behavior, see SI for more details on the thermodynamic approach in the computational details section from page 12), the (001) subsurface cation vacancy (SCV) surface termination and the (111) Fe-tet_1_ termination are expected to be the most stable single crystal surface terminations for {001} and {111} type facets^18–21^.

**Table 3:** Surface (Gibbs) free energies $\gamma$ from DFT calculations and ab initio thermodynamics

Temperature [K]	Gibbs surface free energy $\gamma$ [J/m^2^]			Effective surface free energies ${\gamma }^{* }$ [J/m^2^]		Work of adhesion *W*_adh_ [J/m^2^]	
	(001) DBT	(001) SCV	(111) tet_1_	(001) DBT & (111) tet_1_	(001) SCV & (111) tet_1_	(001) DBT & (111) tet_1_	(001) SCV & (111) tet_1_
0	0.286	−0.217	0.197	−0.050	−0.309	0.247	0.506
450	0.537	0.285	0.487	−0.211	−0.340	0.698	0.827
600	0.628	0.468	0.592	−0.269	−0.351	0.861	0.943
800	0.753	0.718	0.737	−0.350	−0.368	1.087	1.105
	(001)^47^	—	(111)^47^	(001)^47^ & (111)^47^	—	(001)^47^ & (111)^47^	—
0–800	0.96	—	1.09	−0.596	—	1.686	—

An oxygen pressure of 8·10^−^^7^ mbar was assumed. The effective surface free energies (${\gamma }^{* }$) and the work of adhesion (*W*_adh_)(See Methods) are given for NP with the experimental aspect ratios and showing {111} tet_1_ and either {001} DBT or {001} SCV facets. In the last two lines of the table, the surface energy values from Santos-Carballal et al.^47^ and the calculated results for ${\gamma }^{* }$ and *W*_adh_ are presented.

**Fig. 6:**
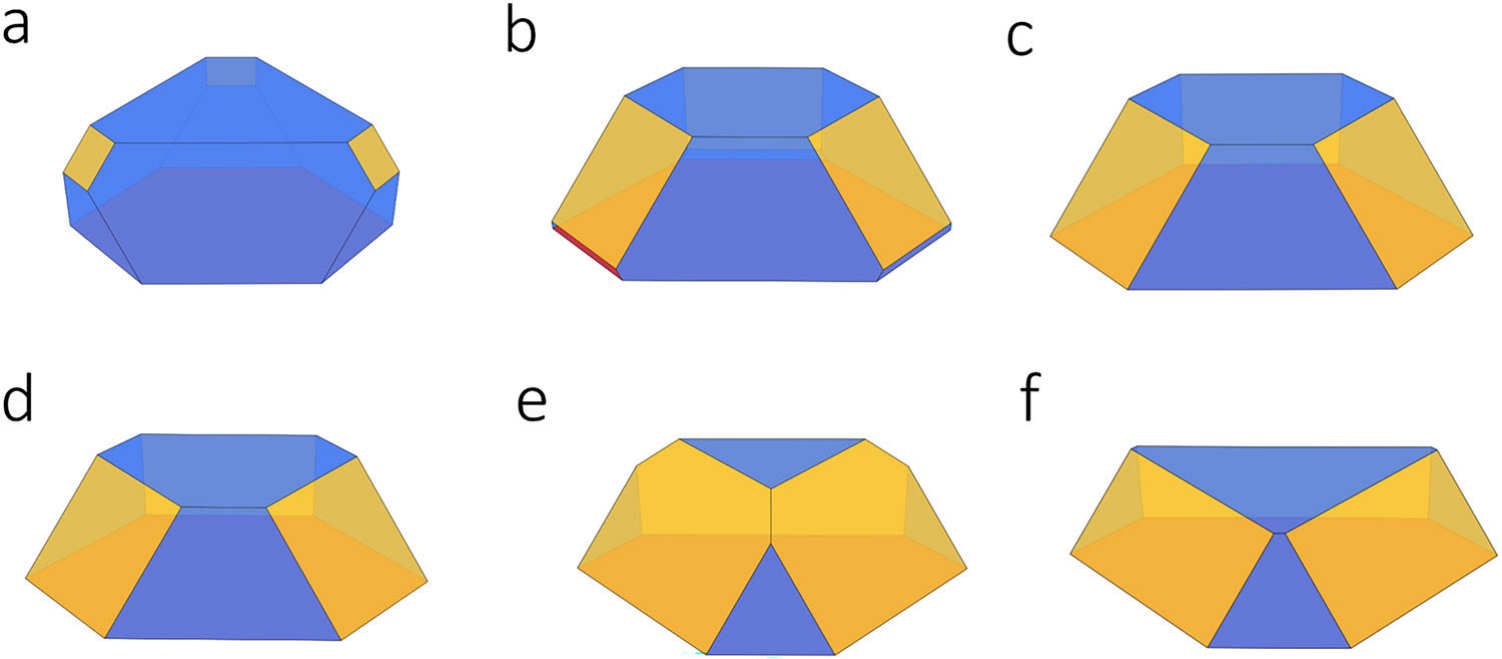
Winterbottom constructions for supported magnetite nanoparticles for an oxygen pressure of p = 8·10^−^^7^ mbar. Given is the shape for calculated surface free energies from this study for {001} DBT and {111} tet_1_ at (**a**) 0 K, (**b**) 450 K, (**c**) 600 K, and (**d**) 800 K. For calculated surface free energies at 600 K (**e**) from this study for {001} SCV and {111} tet_1_ and (**f**) from Santos-Carballal et al.^47^. {111} and {001} facets are shown in blue and yellow, respectively.

Using the SCV energies from Table 3, complete wetting of the Fe_3_O_4_ on the substrate should be observed up to 450 K. However, this was not evident in the experiments. From about 500 K, partial wetting and the formation of well-defined NPs would be expected (see Figure S14). The assumption of an SCV reconstruction for the {001} facets results in significantly reduced {001} surface free energies compared to the respective {111} energies, particularly in the lower part of the experimental temperature range. While a small fraction of {111} facets and dominating {001} facets were predicted at 600 K, the NP shape resembled the DBT results with similar fractions of {001} and {111} facets at 800 K (see Fig. 6 and S14). The values of the facet fractions can be found in Table S6. However, as such drastic changes were not evidenced in the experiments, it seems very unlikely that SCV reconstructions take place in such supported NPs.

Using the surface energies from this study for DBT and tet_1_ terminations for {001} and {111}, respectively, a supported NP shape with strongly dominant {111} facets is predicted at 0 K (see Fig. 6). With increasing temperature at 450 K, 600 K and 800 K, the {111} facets became less dominant while still being the majority facet (see Fig. 6 and Table S6). The NPs adopt a more hexagonal shape, which is in line with the FT-IRRAS results, evidencing the presence of both {111} and {100} type side facets. The experimental observation of larger, nearly triangular NPs may point to a size-dependent shape or kinetic stabilization effects for the shape of larger nanoparticles. Predicting the NP shapes using the surface energies from refs. ^18, 20^ in comparison to our own data yields qualitatively the same results for the NP shape. Meng et al.^46^ predicted the Wulff shape of unsupported iron oxide NPs using surface free energies obtained by a similar computational setup and the same ab initio thermodynamic approach employed here. Their predicted shapes for Fe₃O₄ are in qualitative agreement with our results in Figure S12. However, since the values of the surface energies are not provided in their paper, no predictions are made here based on their data. It is important to note that more facets were studied in ref. ^46^, but the {111} and {001} facets remained the most important for the experimental conditions used in this work. However, for more negative oxygen chemical potentials, the {311} and {110} facets were predicted to appear and dominate the Wulff shapes. Furthermore, Santos-Carballal et al.^47^ predicted the shape of unsupported Fe₃O₄ NPs; however, they did not use a thermodynamic treatment. The shapes of the supported NPs predicted here, based on ref. ^47^, disagree with our experimental results, as described below.

Santos-Carballal et al.^47^ reported the surface energies for (001) and (111) surfaces as 0.96 J/m^2^ and 1.09 J/m^2^, respectively. A tetrahedral Fe-terminated (001) and an octahedral Fe-terminated (111) surface were considered there, which are unfavorable under experimental conditions used in this study compared to DBT, SCV, and tet_1_. Therefore, the surface energies are significantly higher than those in Table 3. The surface free energies derived using their surface models do not depend on the oxygen chemical potential and, consequently, on temperature since stoichiometric models were used. An ab initio thermodynamic investigation was performed on reduced and oxidized surfaces in that study, but the results were not used for NP shape predictions. The shapes of unsupported NPs based on these values and those from Table 3 are discussed in the SI from page 23.

In ref. ^47^, the proposed shape of an unsupported NP was presented (see Figure S11a), while here we use their data for predicting the supported NP shape (see Fig. 6f), which is similar to the shape based on the experimental results. However, the predicted {001} facet fraction (see Table S6) is significantly higher than calculated from our data and than expected from the experiments. This discrepancy between the calculated facet fractions was already expected from the surface energy hierarchies in ref. ^47^ and in our data. Reference ^47^ indicates that the {001} facets have lower surface energies than the {111} facets, while our data predicts the opposite hierarchy (see Table 3). The facet fractions based on data from ref. ^47^ are also in contradiction to the FT–IRRAS results, which suggest a dominance of {111} facets. Furthermore, other Fe_3_O_4_ {001} surface terminations were recently discussed in ref. ^18^. Particularly, the “Fe_oct_ pair” surface model based on ref. ^48^ was proposed to be the stable single crystalline {001} surface at elevated temperatures and just below the typical surface science oxygen chemical potentials. Since this termination would also stabilize the {001} facets compared to {111}, it does not fit to the experimental results either. Consequently, further surface terminations used in refs. ^18, 47^ do not seem to be present on NP systems. Therefore, it is very unlikely that this model allows an accurate description of NP under the given conditions.

In the literature, computational modeling of unsupported cubic magnetite NPs suggested that the {001} facets were not reconstructed, but half of the NP corners showed reconstructions resembling SCV behavior^49,50^. However, these results were obtained for small NPs with a diameter of about 2.3 nm. At such sizes, the effect of corners and edges, as well as other finite size effects, might be more significant compared to the larger NPs in this work. As such size effects, including corner effects, are not considered in the used Wulff/Winterbottom constructions; this cannot be addressed in this study.

The work of adhesion (*W*_adh_) was calculated using Eq. 4 (See Methods) for an additional characterization of the interfaces. The calculated values are shown in Table 3. *W*_adh_ is the energy per unit area to fully separate the NPs from the substrate, assuming no plastic or diffusional modifications. The maximum values calculated here are about 1 J/m^2^. This is significantly smaller than values reported for metal NP on oxide substrates. In the literature, values of 2.0 J/m², 1.9 J/m², and 2.6 J/m² were obtained for Pt NP, Rh NP, and PtRh NP, respectively, all on Al_2_O_3_(0001)^33^. For Pd NP at an Al_2_O_3_ thin film, 2.8 J/m^2^ ^51^, and for Pd  NP at MgO(001), 0.7 J/m^2^ ^52^ were reported. Those values indicate a stronger bond of metal NP to oxide substrates than for the oxide NP investigated here. For oxide–oxide interfaces, values for the *W*_adh_ are more scarce in the literature. For interfaces between ZrO_2_(111) and SiO_2_(0001) slabs^53^, and ZrO_2_(001) and Al_2_O_3_ $(1\bar{1}02)$, the values 2.8 J/m² and 1.4 J/m², respectively, were obtained from DFT calculations. For the former, the authors claim specific local interfacial bonding and rearrangements at the interface to be responsible for the high value and the latter is similar to the values obtained here. Using a Modified Embedded Atoms Method, Ohira et al.^53^ investigated magnetite clusters at various Fe and Cr based oxide surfaces and obtained values of up to 1 J/m^2^, similar to the values obtained here.

The adsorption of formic acid strongly influences the surface energies at 0 K of both facets. For the (111) tet_1_ and the (001) DBT surfaces, the modified surface energies were significantly decreased. The magnitude of the change increased with the coverage. For the (001) surface, the surface energies were reduced by about 0.45 J/m^2^ and 0.9 J/m^2^ for half and full coverage, respectively. The (111) surface energies were reduced by almost the same amount, resulting in NP shapes comparable to those without formic acid. However, a more detailed computational study of the effects of adsorbed molecules and their configurations at the surface, including thermodynamic effects and surface supercells, is beyond the scope of this work.

## Conclusions

We studied magnetite NPs supported by Al_2_O_3_(0001) to gain fundamental insight into the equilibrium shape of supported magnetite nanoparticles. Our GIXRD results combined with SEM images evidenced the growth of epitaxial (111)-oriented magnetite NPs with hexagonal to triangular shapes. The NPs exhibited a height to diameter aspect ratio of 0.42 over a wide growth temperature range, which implies that the NPs possess an equilibrium shape. We observed a size-dependent shape variation with some larger nanoparticles exhibiting a triangular shape, most likely appearing due to kinetic barriers during their formation. Furthermore, we obtained insight into the surface termination of the nanoparticle facets via the adsorption of formic acid as a probe molecule. We demonstrated that polarization-dependent FT-IRRAS is sensitive to the adsorption geometry on the NP side facets. A comparison between the experimental and theoretical results shows that the NP {111} facets are terminated by Fe-tet_1_ ions and the {100} facets are bulk terminated by a mixed oxygen / octahedral Fe layer. No molecular formic acid adsorption species was observed, indicating the high activity of the NPs in reactions with organic acids. Additionally, the NP shapes were predicted via the Wulff/Winterbottom construction using surface free energies from DFT calculations and ab initio thermodynamics. The results are mostly in line with the conclusions drawn from the experiment; however, the stronger {111} side facet FT-IRRAS signal rules out the initial presence of SCV-terminated {100} facets, which would be expected from single-crystal surface results, which become unreconstructed upon formic acid adsorption. Likely, the Wulff/Winterbottom constructions and the accuracy of our calculations come to their limits here. Overall, we have deepened our fundamental understanding into the equilibrium shape of supported magnetite NPs, which can also be utilized to gain a more profound insight into the shape of wet chemically synthesized magnetite NPs and its role in organic molecule adsorption.

## Methods

### Nanoparticle growth

The growth of the NPs and all the analyses were performed at DESY Nanolab^54^. Commercial alumina single crystals, Al_2_O_3_(0001) (miscut < 0.1°), were used as supports. Prior to the growth, the alumina crystals were degassed for 1 h at 773 K in ultra - high vacuum (UHV) with an additional annealing step at 573 K (for 1 h) under 1·10^−^^6^ mbar of atomic oxygen using a thermal cracker to remove carbon impurities from the surface. The growth was carried out from an iron (Fe) rod using an electron beam evaporator. Iron was evaporated at a background pressure of 8·10^−7^ mbar of oxygen. Magnetite NP samples were grown at a rate of ≈ 7 Å/min. Different substrate temperatures of 423 K, 573 K and 773 K (labeled in this work as T1, T2, and T3, respectively) were employed by adjusting the applied current to a filament mounted on the sample holder. The required current corresponding to these temperatures was determined by measuring a set of calibration samples while monitoring the crystal temperature with a mounted thermocouple.

### Morphological and structural characterization

A field - emission scanning electron microscope with a lateral resolution of around 1 nm was used to study the morphology of NPs on the alumina single crystals^54^. The measurements were done in the secondary electron mode at an acceleration voltage of 5 kV using a Through Lens Detector (TLD).

Structural characterization and epitaxy of the NPs were investigated using GIXRD at the incidence angle of 0.3°. Measurements were carried out under atmospheric pressure at room temperature using a six-circle diffractometer at a photon energy of 8.0478 keV (Cu Kα = 1.5406 Å), focused by a parabolic multilayer optics and a hybrid pixel detector with GaAs sensor^54^. Step sizes were adjusted according to the required resolution for each measurement, ranging from 0.05 to 1°.

X-ray reflectivity data were fitted using *Fewlay*, based on Parratt formalism^31,55^. A two - layer model on the alumina substrate was used to fit the data. The model consists of a magnetite layer with free parameters for Gaussian interfacial roughness, thickness, the refractive index components *δ* and *β* (related to the effective electron density profile), and an additional layer to improve the representation of the density profile for deviation from an ideal Gaussian roughness. The error bars of the fitted parameters are within 10%^30^. The average crystalline size of the NPs (D) over size distribution was estimated using^37^:

2 $$D=\frac{2{{\rm{\pi }}}}{\varDelta Q}\,$$

where ΔQ is the full-width half maximum of the Bragg peak in momentum space determined by fitting a Gaussian to the experimental line profile^37^.

### Spectroscopic characterization by XPS and FT-IRRAS

After the growth, the NP samples were directly transferred to an XPS chamber under UHV conditions. Room temperature XPS measurements were performed at a photon energy of 1486.6 eV using a monochromatized Al Kα source. A flood gun was used for the XPS measurements on the NP samples. The measurements were carried out at a pressure of 5·10^-10^ mbar. Adsorption of formic acid on the NPs at room temperature was studied using a UHV chamber with a base pressure of 8·10^−10^ mbar. The chamber was connected to a liquid-nitrogen-cooled mercury-cadmium-telluride (MCT) detector via differentially pumped potassium bromide (KBr) windows. The NP samples were transferred from the growth chamber to the IR chamber under UHV conditions. Prior to the transfer, the NP samples were flash annealed to 393 K (10–15 min) to desorb contaminant adsorbates from the samples^56,57^. Formic acid with a purity of > 99% was further purified by multiple freeze-pump-thaw cycles and exposure was done stepwise into the chamber through a leak valve. FT-IRRAS spectra were recorded in *s* and *p* polarization modes with a resolution of 2 cm^−1^ after dosing 0.2, 1, 5, and 10 L (Langmuir) of formic acid, dosing pressures of 1·10^−9^, 1·10^−8^ and 1·10^−7^ mbar, respectively.

### Nanoparticle shape prediction

The thermodynamic equilibrium shape of (nano-)particles can be obtained from the surface free energies of the relevant facets using the Wulff construction^58,59^. When the particles are in contact with a substrate, the effect of the particle-substrate interaction is considered in the Winterbottom construction^60^. Both approaches rely on surface energies to predict the NP shape. For the Winterbottom construction, the effective surface energy between the substrate and the NP must also be considered, which is the difference between substrate-NP interface energy and the substrate’s surface energy (see also Eq. 3). The Wulff construction and related approaches have inherent limitations. For example, the formation energies of corners and edges are not considered, which become more relevant as particles become smaller. Additionally, strain relaxation effects are not considered.

Here, surface energies for the dominant (001) and (111) magnetite surface facets were calculated using density functional theory (DFT) based on previous publications^23,26,50^. In addition, DFT calculated surface energies from the literature were used for comparison^18,20,47^. For all sources, the DFT calculations were performed with the Vienna Ab initio Simulation Package (VASP, here version 5.4.4)^61–64^. The computational details of our own calculations can be found in the SI in the computational details section from page 9. The images of the atomic configurations in Fig. 1 and S10, as well as the schematic model of the NPs in Fig. 4b, were created employing VESTA^65^.

Our own DFT results and some of the literature data^18^ use the Distorted Bulk Truncation (DBT) surface model for the (001) surface, where the surface is terminated by a layer of oxygen and octahedral Fe atoms, while for the (111) surface, a tetrahedral-Fe terminated surface (Fe-tet_1_) is used. For (001), the subsurface cation vacancy (SCV) reconstruction^19^ is stable for typical UHV conditions; however, adsorption, e.g., hydrogen or formic acid, can lift the reconstruction and a DBT surface is recovered^23–25^. These terminations are considered to be the most favorable for clean and adsorbate-covered surfaces for the experimental growth conditions used in this work^19–21,23,26^ (the atomic configurations of two prototypical (001) DBT (Supplementary Data 1) and (111) tet1 (Supplementary Data 2) surfaces are supplied in the Supporting Information as data files in the VASP POSCAR format). However, the results of Santos-Carballal et al.^47^ were calculated for a tetrahedral and octahedral Fe-terminated (001) and (111) surface, respectively, whereas DBT, SCV and tet_1_ were not investigated in that study.

Compared to our published results^23,26^, for this study, thicker slabs up to 25 layers were added and symmetry constraints in the DFT calculations were removed to reproduce physically expected results on atomic charges and corresponding oxidation states following our recent work^27,50^. Results from these DFT calculations for (001) DBT and (111) tet_1_, including the relaxed atomic configurations, Bader charges, derived oxidation states and magnetic moments are given in the SI (see Tables S4 and S5). Surface energies were obtained using the “direct” approach as the difference between slab and bulk energies from DFT calculations. For the clean, adsorbate-free (001) and (111) surfaces, slabs of up to 25 and 23 layers, respectively, were used. The clean (001) surface was modeled using a √2×√2 R45° surface supercell to accommodate DBT and SCV surfaces. A 2 × 2 surface supercell was used for the clean (111) surface. This cell is energetically more favorable than a 1 × 1 cell because finite-size effects result in unfavorable charge distributions in the smaller cell^50^. For the adsorbate-covered surfaces, the most favorable adsorption structures from Arndt et al.^23^ and Creutzburg et al.^26^ were used for the assessment of the (001) and (111) surfaces, respectively. Details on the adsorption structures, including preferred binding sites and motifs, are summarized in the SI in the computational details section on page 12.

The surface energies from the DFT calculations are based on the internal energies at 0 K. To approximate experimentally relevant conditions (temperatures from 450 to 800 K and 8·10^−^^7^ mbar oxygen pressure), ab initio thermodynamics^44^ was used to estimate the surface free energies at finite temperature and experimental partial pressures, here during the growth process. Further details can be found in the SI from page 12. The surface free energies were calculated for 450, 600 and 800 K, slightly off the experimental growth conditions, due to the availability of thermochemical data^66^.

Using the surface free energies at different temperatures and for the experimental oxygen pressures, the unsupported and supported nanoparticle shapes were estimated by the Wulff construction and the Winterbottom construction, respectively, using the WulffPack code^45^. The constructions and various computational approaches and tools are nicely summarized in a review by Boukouvala et al.^59^. The Wulff construction is based on surface energies, while for the Winterbottom construction, additionally, the effective surface free energy (${\gamma }^{* }$) is essential. This quantity is defined as the difference between the interface energy (${\gamma }_{{{\rm{interface}}}}$) and the substrate surface free energy (${\gamma }_{{{\rm{substrate}}}}$).

DFT calculations on the interface between the substrate and the NP would be computationally very intensive and particularly finding the most favorable interface structure is challenging. To minimize the computational cost and resource consumption, such interfaces were not explicitly calculated; however, the effective surface free energy (${\gamma }^{* }$) was derived from the experimentally observed aspect ratios of the supported nanoparticles and the calculated magnetite surface free energies. The effective surface free energy can be obtained by the following equation^51^:

3 $${\gamma }^{* }={\gamma }_{{{\rm{interface}}}}-{\gamma }_{{{\rm{substrate}}}}=\sqrt{\frac{3}{2}}\frac{h}{w}{\gamma }_{100}-{\gamma }_{111\,}$$

with the nanoparticle height $(h)$, the nanoparticle width $(w)$ and the surface energies ${\gamma }_{100}$ and ${\gamma }_{111}$ for the (100) and (111) facets, respectively. The average experimental aspect ratio of $\frac{h}{w}=0.42$ was used for simplicity, as the variations in the experiments were found to be small. For effective surface energies greater and less than zero, the height of the supported NP will be more and less than half of the height of the unsupported NP. The range of reasonable effective surface free energy values is limited by the boundary scenarios of complete wetting and no wetting of the NP on the substrate^60^.

In the current work, the effective surface free energy between the NP and the substrate (see below) becomes negative (see Table 3). The main consequence is that the height of the supported NP is less than half of the height of the corresponding unsupported NP. The WulffPack code, however, could not handle negative effective surface energies up to version 1.3. Therefore, we have added this functionality employing the Closest Vertex technique for obtaining the NP shape from the surface energies as it is, e.g., implemented similarly in the Wulffmaker code^67^. The computational runtime of this method scales like *O*(*N*^4^), where N is the total number of facet normals. Thus, it is significantly slower than the Dual Wulff Shape approach, typically scaling like *O*(*N* log *N*) so far used in WulffPack. Therefore, the Closest Vertex Technique is only used in case of negative effective surface energies. However, it has to be added that the runtimes for all tested scenarios are in the order of a few seconds or below and thus not a major issue here. Our addition has already been merged into the source code of WulffPack by the developers and has been available in WulffPack since version 1.4^68^.

The work of adhesion (*W*_adh_)^51^ was calculated using the Dupré equation and Eq. 3:

4 $${W}_{{adh}}={\gamma }_{111}+{\gamma }_{{{\rm{substrate}}}}-{\gamma }_{{{\rm{interface}}}}={\gamma }_{111}-{\gamma }^{* }=2\,{\gamma }_{111\,}-\sqrt{\frac{3}{2}}\frac{h}{w}{\gamma }_{100}\,$$

## Supplementary information

Supporting Information

Description of Additional Supplementary Files

Supplementary Data 1

Supplementary Data 2

## Data Availability

The data that support the findings of this study are available from the corresponding authors upon reasonable request.
