# Supplementary material for: Equilibrium shape and surface termination of supported magnetite nanoparticles
Source: Commun Chem. 2026 Apr 11;9:158. doi: 10.1038/s42004-026-02008-4 (PMC13083894; doi:10.1038/s42004-026-02008-4)
Supplement: Supplementary file 1 — Supporting Information [file 42004_2026_2008_MOESM1_ESM.pdf]

Supplementary Information: Equilibrium Shape and Surface Termination of Supported Magnetite Nanoparticles

Mohammad Ebrahim Haji Naghi Tehrani[a,b], Daniel Silvan Dolling[a,b], Jan-Christian Schober[a,b], Esko Erick Beck[a,b], Mona Kohantorabi[a], Arno Jeromin[a], Ludwig J.V. Ahrens-Iwers[c,d], Thomas F. Keller[a,b], Vedran Vonk[a], Gregor B. Vonbun-Feldbauer[c,d], Heshmat Noei[a], Andreas Stierle[a,b*]*

a Centre for X-ray and Nano Science (CXNS), Deutsches Elektronen-Synchrotron (DESY), 22607 Hamburg, Germany

b Physics Department, University of Hamburg, D-20355 Hamburg, Germany

c Institute for Interface Physics and Engineering, Hamburg University of Technology, 21073 Hamburg, Germany

d Institute of Surface Science, Helmholtz-Zentrum Hereon, 21502 Geesthacht, Germany

Emails: heshmat.noei@desy.de; andreas.stierle@desy.de

Scanning electron microscopy and image analysis

Secondary electron SEM images were collected as described in the Methods section of the main manuscript. To extract the nanoparticle size distribution from the SEM images, image analysis was applied using the imagej-based image processing program Fiji[1]. For an automated nanoparticle identification and size determination, the routine "analyze particle" of Fiji was employed, with several steps of preprocessing the SEM image involved. These steps include i) cropping a suitable region of interest (ROI), ii) scaling the pixel images with the scale marker in the SEM image resulting in a pixel size of $1.04 \times 1.04\ nm^2$, iii) smoothening to increase the image quality for the particle analysis, and vi) binarization via the auto local thresholding algorithm MidGrey, which chooses the mean of the minimum and maximum grey values in the local window. The resulting binarized SEM image was used for the particle analysis using the Fiji "Analyze Particles" routine. Particles located partially outside the considered ROI are disregarded. The error is estimated to correspond to the size of around 2 pixels. Particles with pixel sizes in the range of the error may be underweighted in the histogram.

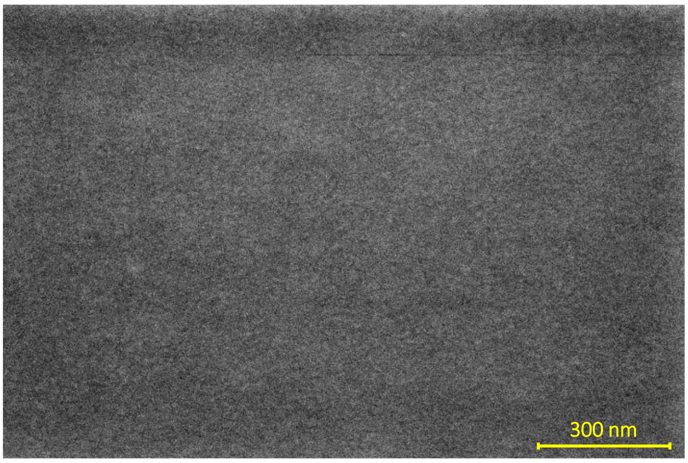

Figure S1. SEM image of magnetite nanoparticles (NPs) after growth at 423 K (T1) on an $Al_2O_3(0001)$ substrate.

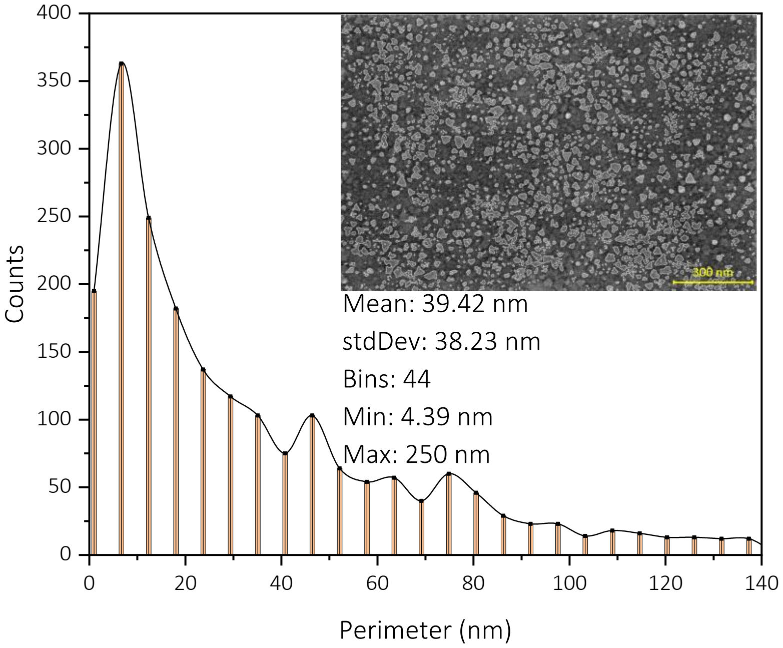

Figure S2. Binned counts of particle sizes obtained from SEM analysis of the T3 sample. Inset: overlay SEM image showing the detected nanoparticles (counts for perimeters larger than 140 nm are all less than 25).

Table S1. XRR fit parameters of the magnetite NPs grown at 423, 573, and 773K

	T1	T2	T3
d_2 (Å)	-	3.00	3.40
σ_2 (Å)	-	2.94	6.24
$2\delta_2$	-	6.39E-06	6.73E-06
$2\beta_2$	-	1.00E-09	2.18E-08
$d_{Magnetite\ layer}$ (Å)	44.82	41.15	41.64
$\sigma_{Magnetite\ layer}$ (Å)	5.72	14.85	16.98
$2\delta_{Magnetite\ layer}$	3.21E-05	3.14E-05	3.69E-05
Coverage$_{Magnetite\ NPs}$ (%)	72.26	72.35	83.36
$2\beta_{Magnetite\ layer}$	1.03E-09	4.12E-07	5.18E-09
$\sigma_{sapphire}$ (Å)	3.66	6.09	2.42

**Theoretical values*
$2\delta_{Magnetite}$= 4.66E-5
$2\beta_{Magnetite}$= 4.26E-6

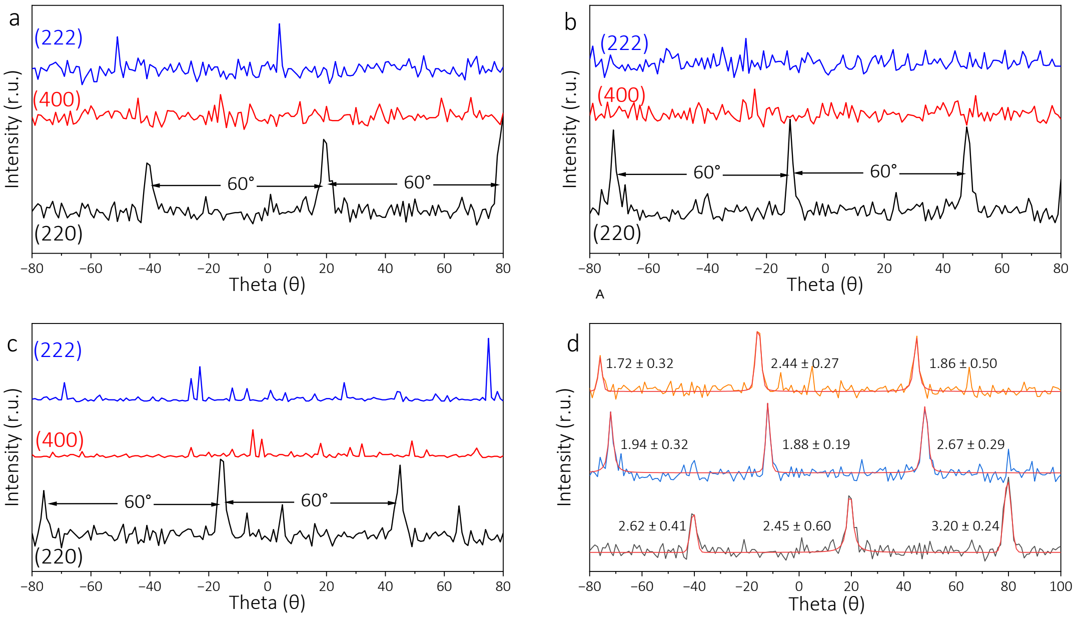

Figure S3. Azimuthal (in-plane) diffraction patterns in search of (222), (400), and (220) reflections of magnetite: a) T1, b) T2 and c) T3. d) Fitted peak analysis of the (220) rocking scans measured on T1 (black line), T2 (blue line) and T3 (orange line); the values indicate the FWHM of the peaks estimated from the fits.

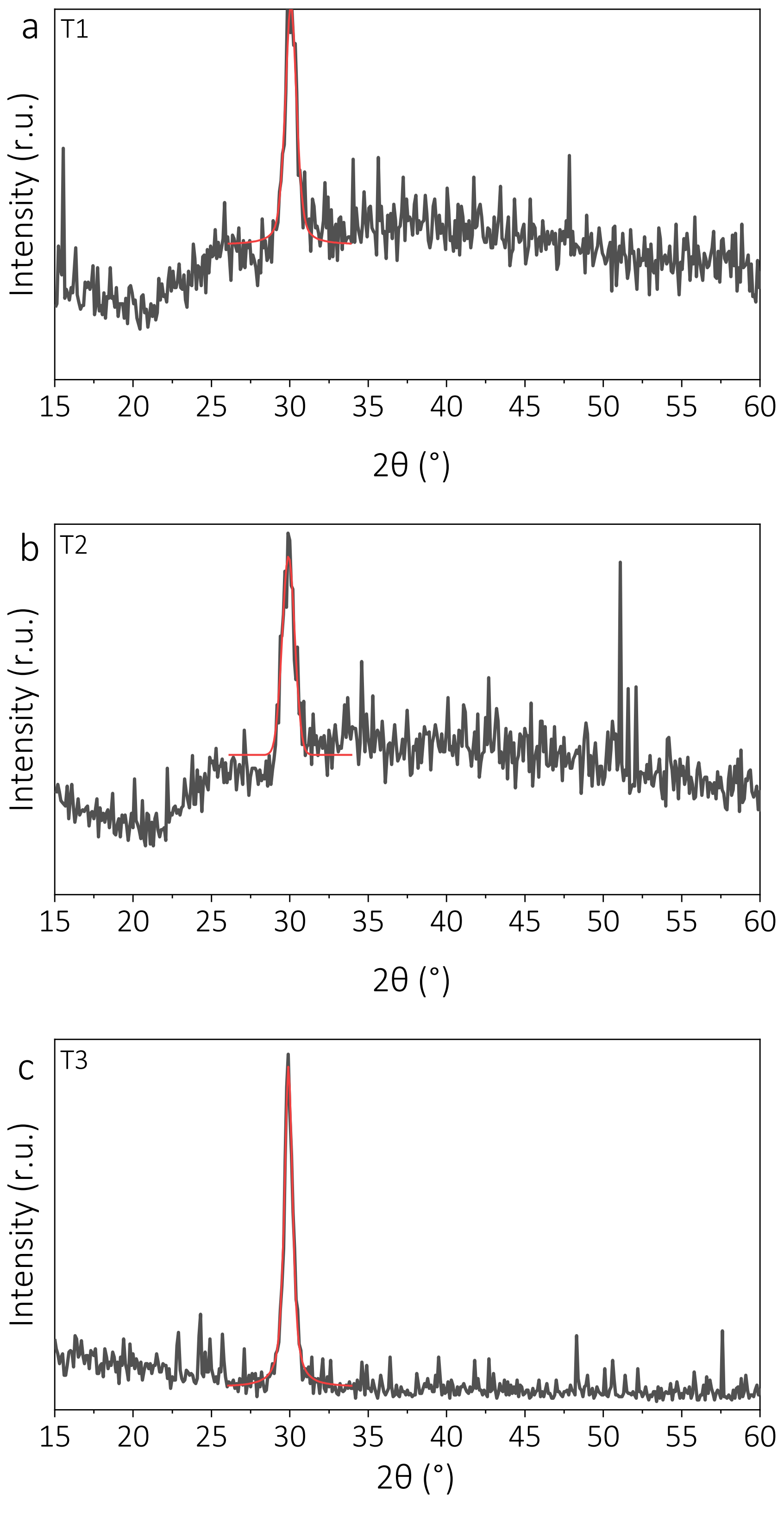

Figure S4. In-plane radial scans in the alumina [$10\bar{1}0$] direction measured for magnetite NPs grown at 423, 573 and 773 K (a-c). Fitted curves are shown with red lines.

Table S2. Calculated in-plane lattice parameter (*a*) and d-spacing of the nanoparticles compared to magnetite and maghemite bulk crystal structures

	T1	T2	T3	Magnetite[a]	Maghemite[b]
2θ	30.04	29.89	29.91	-	-
a (Å)	8.41	8.45	8.44	8.396	8.351
d_{220} (Å)	2.972	2.987	2.985	2.967	2.953
d_{440} (Å)	-	-	1.489	1.484	1.476

[a] JCPDS file No. 39-1346.

[b] JCPDS file No. 19-629.

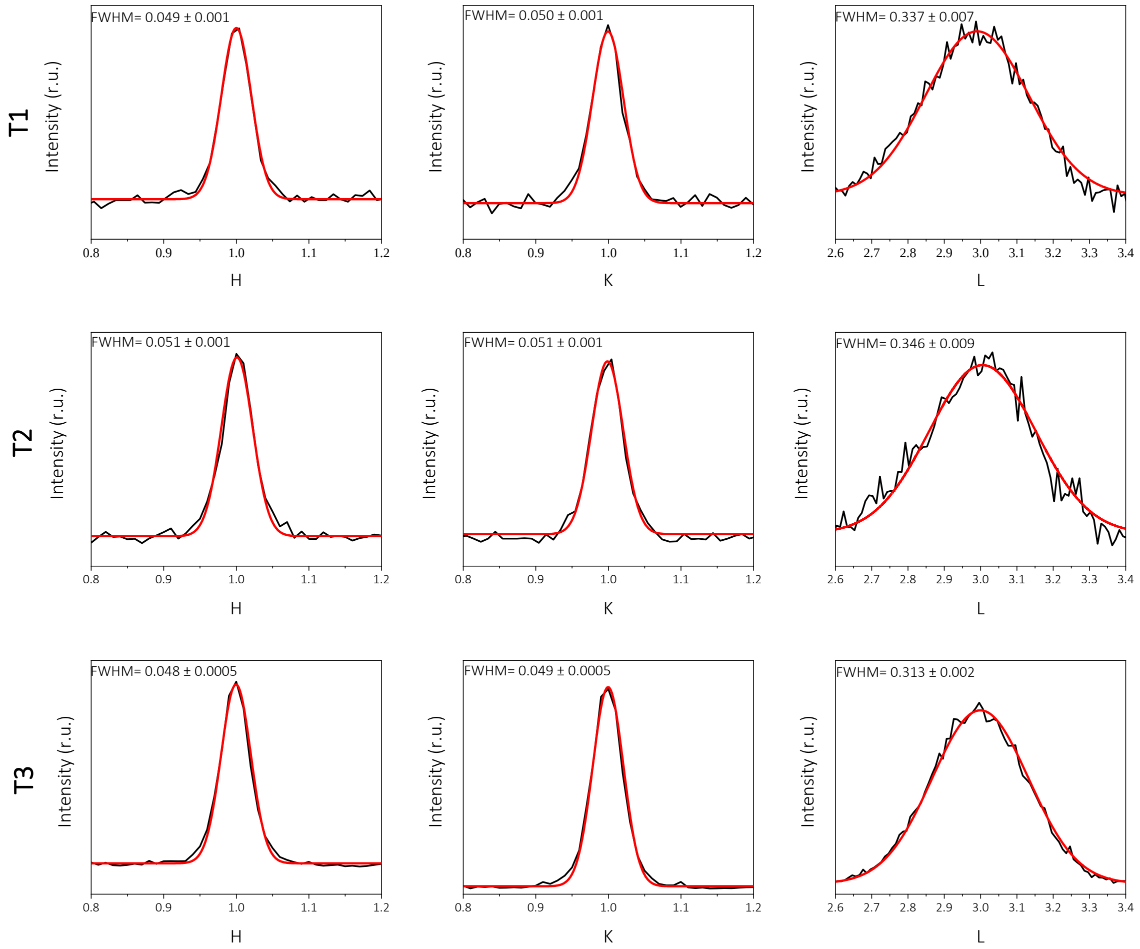

Figure S5. Line scans in h, k, and l directions through the (113) Bragg peak (identical to the $(3\bar{1}1)$ reflection in bulk coordinates) of the magnetite NPs grown at 423, 573 and 773 K (black lines: measured data, red lines: fit curves).

Table S3. Average size determination based on the line scans through the (113) Bragg peak of the NPs. The bulk structure unit cell [α = β = γ = 90°, a = b =c] with a lattice constant of a = 8.396 Å[2] was converted to a hexagonal (111) surface unit cell using $\alpha_s = \beta_s = 90°$, $\gamma_s = 120°$ and $a_s = b_s = \frac{a}{\sqrt{2}}, c_s = a \times \sqrt{3}$ as the lattice parameters

	Diameter *D* (Å)	Height *H* (Å)	*H/D*
T1	104	43	0.413
T2	101	42	0.416
T3	106	47	0.443

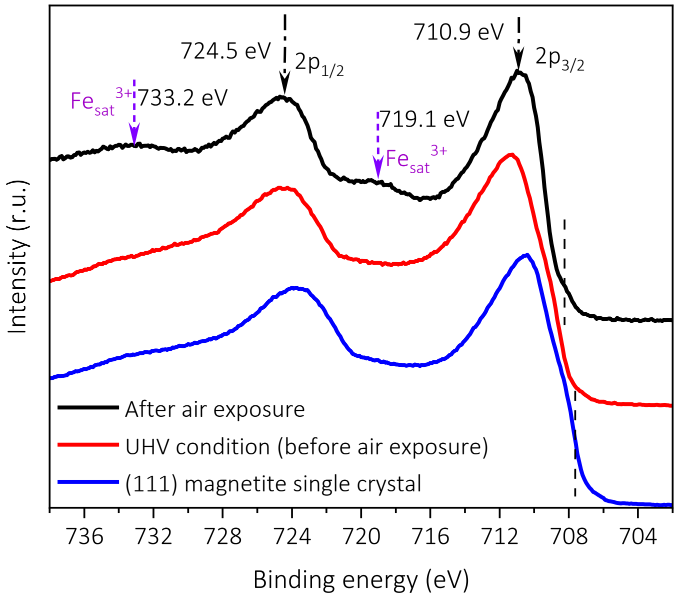

Figure S6. Fe 2p core level spectra of the T3 sample recorded in UHV and after exposure to air, a spectrum of magnetite (111) single crystal sample is shown for comparison.

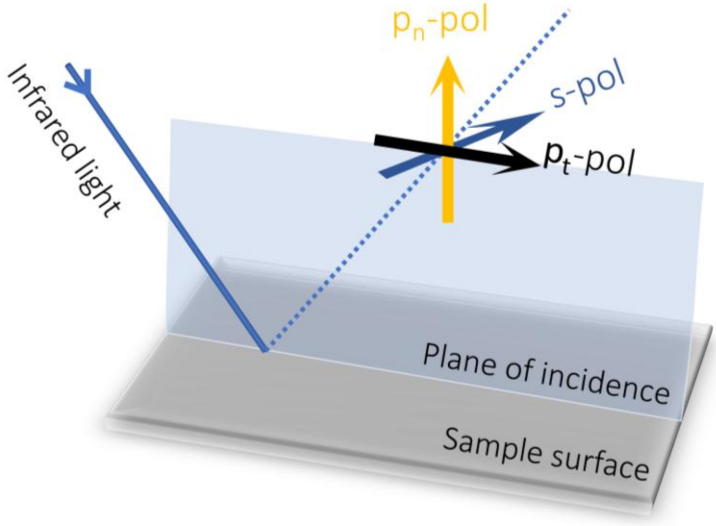

Figure S7. *s* and *p*-polarized light components in IRRAS with respect to the plane of incidence.

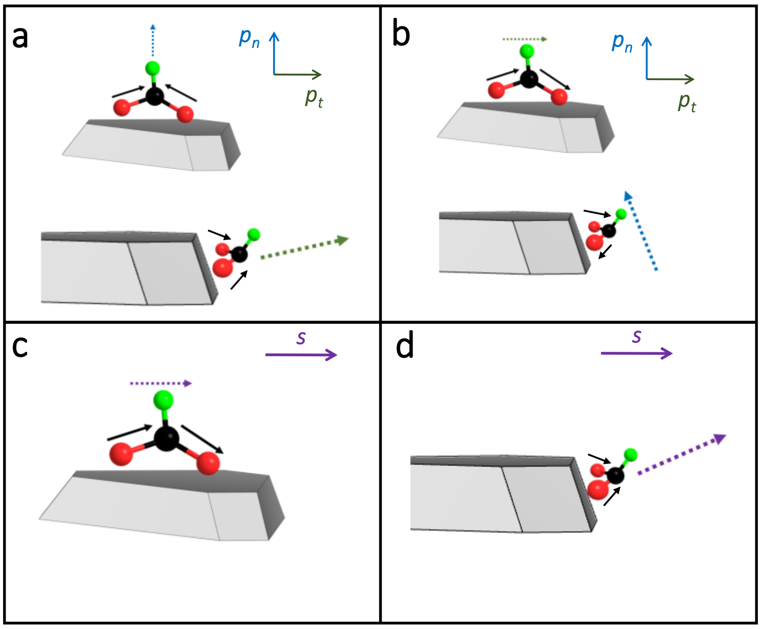

Figure S8. a) schematic models representing the symmetric dynamic dipole moments (dashed arrows) of formate on the top and side (111) facets excited by the *p*-pol light, b) schematic models representing the asymmetric dynamic dipole moments of formate on the top and side (111) facets excited by the *p*-pol light, c) schematic model representing the asymmetric dynamic dipole moment of formate on the top (111) facet of the NP excited by the *s*-pol light, d) schematic model representing the symmetric dynamic dipole moment of formate on the side (111) facet excited by the *s*-pol light.

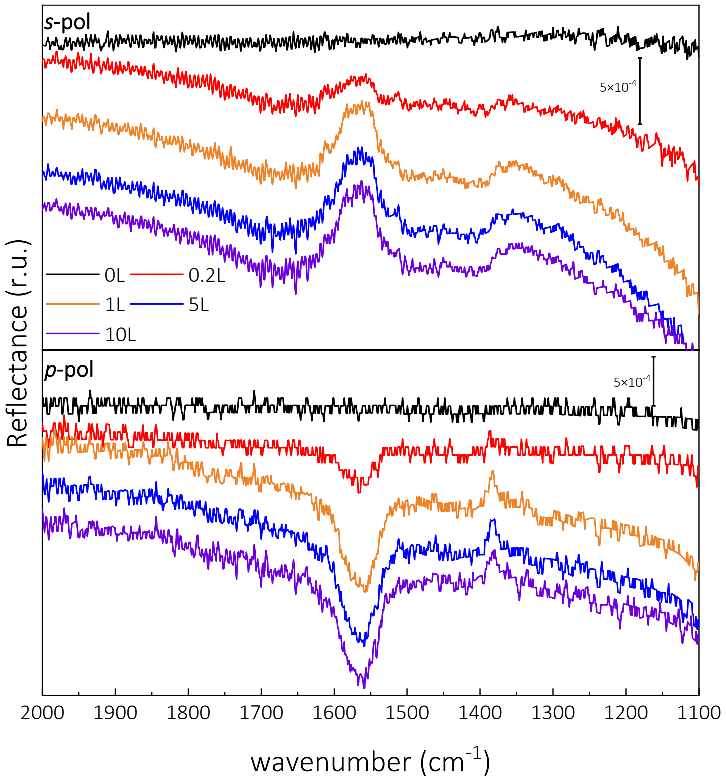

Figure S9. *s* and *p*-polarized IR spectra recorded at different dosing steps of formic acid on magnetite NPs grown at 423K (T1).

Computational Details

Spin-polarized Density Functional Theory (DFT) calculations were performed following the setup used in our previous studies[3-5]. The computational setup was chosen to allow for comparison with data from those previous studies. Moreover, the setup presents a good compromise between computational cost and accuracy for predicting different properties of Fe_3O_4[6]. The Vienna Ab Initio Simulation Package (VASP, version 5.4.4)[7-10] with the PBE exchange correlation (XC) functional[11], the Hubbard U approach in the Dudarev approximation[12] (U_{eff} = 4 eV on Fe d-electrons), and Projector Augmented Wave (PAW) pseudopotentials[13] was used to study magnetite bulk and surfaces. Convergence with respect to

k-point sampling was achieved within 1meV/atom by using k-point grids of 11 × 11 × 11 and 5 × 5 × 1 for the bulk ($Fd\bar{3}m$, 56 atoms) and surface supercells used, respectively. An energy cut-off of 520 eV was chosen to converge the surface energies within 1 mJ/m^2 and the total energies within 3 meV/atom. Details on convergence tests can be found in Appendix B of Sellschopp[14]. Gaussian smearing of 0.05 eV was used for all relaxations. Structural optimizations were performed stepwise with a fixed cell shape using the conjugate gradient algorithm until convergence was achieved within 1 × 10^{-6} eV and 2 × 10^{-6} eV in energy for bulk and surface calculations, respectively, and within 5 meV/Å in force. Additional static calculations on the optimized structures were performed using the tetrahedron method with Blöchl corrections[15] and tighter energy convergence criteria of 1 × 10^{-8} eV and 2 × 10^{-8} eV for bulk and surface calculations, respectively, to obtain more accurate total energies. For the bulk calculations, the optimized lattice constant of 8.48 Å was used. For surface calculations, the bulk lattice constant was employed parallel to the surface, while allowing the slabs to relax normal to the surface. Dipole corrections[16] perpendicular to the surface were used for all surface calculations. The surfaces were modeled using symmetric periodic slabs separated by a vacuum region of 23 Å. The oxidation states of the Fe atoms can be assigned, e.g., based on atomic charges from Bader charge analyses[17]. Bader charge analyses were performed here using the implementation of Henkelman *et al.*[18-20] and following the distinction of Fe^{2+} and Fe^{3+} for Fe_3O_4 from Ref.[21]. The results are presented below in Tables S4 and S5. The oxidation states for model systems used here were discussed in previous publications in more detail[5,22]. The magnetic moments follow the trends that are expected for ferrimagnetic magnetite, particularly, the systems were checked to ensure that no unphysical spin flips had occurred (see Tables S4 and S5).

Surface energies were obtained using the "direct" approach as the difference between slab and bulk energies from DFT calculations at T = 0 K. For magnetite, the following equation can be used:

$$E_{\mathrm{surf},Fe_3O_4} = \frac{1}{2A}\left(E_{\mathrm{slab}} - \frac{N_{\mathrm{Fe}}\,E_{Fe_3O_4}^{bulk}}{3} - \left(N_{\mathrm{O}} - \frac{4}{3}N_{\mathrm{Fe}}\right)\frac{E_{O_2}}{2}\right) \qquad \mathrm{Eq.\,S1}$$

E_{surf} is the surface energy. E_{slab} is the total energy of the slab, while E_{bulk} is the total energy of one formula unit of the bulk. E_{O_2} gives the total energy of an isolated oxygen molecule. The single O_2 molecule was placed in a large orthorhombic unit cell with lattice constants > 20 Å to minimize interactions with its periodic images and to allow for physically reasonable occupation numbers of the electronic p-orbitals in a spin-polarized calculation. N_{O} and N_{Fe} represent the number of O and Fe atoms, respectively, in the surface slab. *A* is the area of the surface. The factor ½ accounts for the two surfaces of the slab model. For the stoichiometric Fe_3O_4 slabs, the last term in Eq. S1 becomes zero.

As this approach tends to produce fluctuating energies with thickness, several slab thicknesses were tested in our work. For the (001) surface, the average of surface energies of slabs consisting of 9 layers (~8.2 Å), 17 layers (~16.7 Å) and 25 layers (~25.2 Å) were used, while for the (111) surface, slabs consisting of 11 layers (~7.7 Å) and 23 layers (~17.5 Å) were used. Atomic models of the two surfaces are presented in Figure S10. For the (001) surfaces, two alternating types of layers are observed, consisting either of octahedral Fe and O atoms, or of only tetrahedral Fe atoms (see Figure S10a). For (111) surfaces, each layer contains only one type of atoms, namely tetrahedral Fe, octahedral Fe and O (see Figure S10b). The fluctuations of the surface energies for a single facet were found to be about 10 meV/Å^2 and significantly smaller than the difference between the two facets of about 100 meV/Å^2 at 0 K.

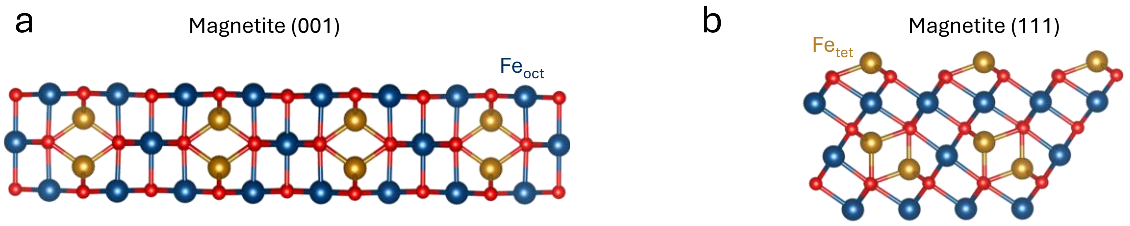

Figure S10. Surface structures of magnetite a) (001) and b) (111).

For the adsorption of formic acid, the following surface models from Arndt *et al.*[3] and Creutzburg *et al.*[4] were used. For the (001) surface, a √2×√2 R45° surface supercell with the DBT (distorted bulk truncated) termination was used, since formic acid adsorption lifts the subsurface cation vacancy (SCV) reconstruction[3]. Formate adsorbs preferentially in bridging bidentate geometries at the (001) surface (see Figure 1). Half and full coverage refer here to one and two formate adsorbates, respectively, per surface supercells. Full coverage is defined here as each surface Fe atom is bonded to one carboxy oxygen. For the (111) surface, a √3×√3 R30° surface supercell with a tetrahedral iron ($Fe\text{-}tet_1$) termination was used to allow for more favorable adsorption structures where three formate adsorbates form triangular structures. The adsorbates exhibit either quasi-bidentate or chelating geometries (see Figure 1) since bidentate is geometrically not possible because of the distance of the tetrahedral iron neighbors at the surface. As mentioned in the manuscript, the results for adsorption should only be seen as indicators for the trend, while a full quantitative analysis of the effect of adsorbates would warrant a more detailed study, which is beyond the scope of this manuscript.

Ab initio thermodynamics[23] was used to estimate the surface free energies ($\gamma_{\mathrm{surf},Fe_3O_4}$). Details on the specific application of this method to these magnetite surfaces can be found in previous papers and their supporting information[3,4]. Here, the change of the oxygen chemical potential ($\Delta\mu_{O_2}$) is used as a variable that can be translated into pressures (p) at given temperatures (T), assuming ideal gas behavior and based on thermochemical data[24] for $\Delta\mu_{O_2}(p^0, T)$ via:

$$\Delta\mu_{O_2}(p_{O_2},T) = \Delta\mu_{O_2}(p^0,T) + k_B T\, ln\left(\frac{p_{O_2}}{p^0}\right), \qquad \text{Eq. S2}$$

where p^0 is the standard pressure of 1 bar. Eq S1 adapts in the following way:

$$\gamma_{\mathrm{surf},Fe_3O_4} = \frac{1}{2A}\left(E_{\mathrm{slab}} - \frac{N_{\mathrm{Fe}}\, E^{bulk}_{Fe_3O_4}}{3} - \left(N_{\mathrm{O}} - \frac{4}{3}N_{\mathrm{Fe}}\right)\frac{1}{2}\left(E_{O_2} + \Delta\mu_{O_2}\right)\right) \qquad \text{Eq. S3}$$

For formic acid, additional terms as for oxygen have to be added to Eq. S3 and an isolated monomer was used as a reference since the NPs were grown well below the monomer-dimer transition pressure of about 10^{-3} bar formic acid partial pressure. The mixing entropy is neglected here.

The calculated O_2 energy affects the surface (free) energies of non-stoichiometric systems (see Equations S1 and S3). Calculating this energy accurately using DFT is challenging and depends on the chosen XC functional (see, for example, Ref.[25]). Errors in the oxygen energy affect the surface energies of the studied facets differently, as the impact depends on the number of off-stoichiometric atoms, which varies among the surface models investigated. For instance, a decrease of the O_2 energy by 0.3 eV results in a change of 90, 40, or 50 meV in the surface energies of (001) SCV, (001) DBT, and (111) tet-1, respectively. For DBT and tet-1 surfaces, the changes are very similar, resulting in only minor changes to the shape of NP exhibiting these surfaces under relevant experimental conditions. Corresponding facet fractions change by roughly 1-2%. For SCV and tet-1, however, the difference of the changes is larger. Thus, the facet fractions are changed by up to 4%. The NP shapes remain qualitatively largely unaffected. However, the prediction of complete wetting for SCV at lower temperatures might be reversed. Nevertheless, SCV does not seem to occur at the NPs anyway. A qualitative change could occur if the energetic hierarchy of the surfaces were affected. For the lower temperatures investigated in this study, this would only occur at oxygen chemical potentials far below the relevant region. Such transitions approach the relevant oxygen pressures only at elevated temperatures (800 K),

especially considering the DBT vs. SCV stability. This could explain why DBT occurs at high temperatures. However, since SCV does not seem to occur at NPs at lower temperatures where it is favorable for single crystalline surfaces, errors in O_2 energy do not seem to play a significant role here.

DFT results

Calculated Bader charges and derived oxidation states, as well as magnetic moments from the DFT calculations for representative (001) and (111) surface models, are shown in Tables S4 and S5. The atomic configurations of two prototypical (001) DBT (Supplementary Data 1) and (111) tet1 (Supplementary Data 2) surfaces are supplied in the SI as data files in the VASP POSCAR format.

Table S4. Calculated magnetic moments, Bader charges and oxidation states derived from the charges from the DFT calculations for a DBT Fe_3O_4 (001) slab with 25 atomic layers and a √2×√2 R45° surface unit cell. Each line represents one atom in the calculated system. The data is presented in blocks of the tetrahedral irons Fe (tet), the octahedral irons Fe (oct), and the oxygens O. The first column provides a numeric label for each atom, the second numbers the atomic layer in which the specific atom is located, the third gives the element and configuration for Fe, the fourth the Bader charge, the fifth the relative atomic charge as the difference between the element specific number of valence electrons used in the DFT calculations (8 for Fe, 6 for O) and the Bader charge, the sixth the oxidation states derived from the relative atomic charge and seventh the magnetic moment. Charges are given in elementary charges (q) and magnetic moments in Bohr magnetons (μ_B)

#	Layer	Element	Electronic Bader charge (-q)	Relative atomic charge (q)	Oxidation State	Magnetic Moment (μ_B)
1	2	Fe (tet)	6.32	1.68	3+	-4.09
2	2	Fe (tet)	6.32	1.68	3+	-4.09
3	4	Fe (tet)	6.36	1.64	3+	-4.05
4	4	Fe (tet)	6.36	1.64	3+	-4.05
5	6	Fe (tet)	6.37	1.63	3+	-4.07
6	6	Fe (tet)	6.37	1.63	3+	-4.07
7	8	Fe (tet)	6.36	1.64	3+	-4.06
8	8	Fe (tet)	6.36	1.64	3+	-4.06

9	10	Fe (tet)	6.35	1.65	3+	-4.06
10	10	Fe (tet)	6.35	1.65	3+	-4.06
11	12	Fe (tet)	6.37	1.63	3+	-4.06
12	12	Fe (tet)	6.37	1.63	3+	-4.06
13	14	Fe (tet)	6.37	1.63	3+	-4.06
14	14	Fe (tet)	6.37	1.63	3+	-4.06
15	16	Fe (tet)	6.36	1.64	3+	-4.06
16	16	Fe (tet)	6.36	1.64	3+	-4.06
17	18	Fe (tet)	6.37	1.63	3+	-4.06
18	18	Fe (tet)	6.37	1.63	3+	-4.06
19	20	Fe (tet)	6.36	1.64	3+	-4.07
20	20	Fe (tet)	6.36	1.64	3+	-4.07
21	22	Fe (tet)	6.36	1.64	3+	-4.05
22	22	Fe (tet)	6.36	1.64	3+	-4.05
23	24	Fe (tet)	6.32	1.68	3+	-4.09
24	24	Fe (tet)	6.32	1.68	3+	-4.09
25	1	Fe (oct)	6.35	1.65	3+	4.18
26	1	Fe (oct)	6.35	1.65	3+	4.18
27	1	Fe (oct)	6.35	1.65	3+	4.18
28	1	Fe (oct)	6.35	1.65	3+	4.18
29	3	Fe (oct)	6.35	1.65	3+	4.14
30	3	Fe (oct)	6.34	1.66	3+	4.16
31	3	Fe (oct)	6.34	1.66	3+	4.16
32	3	Fe (oct)	6.35	1.65	3+	4.14
33	5	Fe (oct)	6.62	1.38	2+	3.65
34	5	Fe (oct)	6.62	1.38	2+	3.65
35	5	Fe (oct)	6.62	1.38	2+	3.65
36	5	Fe (oct)	6.62	1.38	2+	3.65
37	7	Fe (oct)	6.35	1.65	3+	4.14
38	7	Fe (oct)	6.35	1.65	3+	4.15
39	7	Fe (oct)	6.35	1.65	3+	4.15
40	7	Fe (oct)	6.35	1.65	3+	4.14
41	9	Fe (oct)	6.65	1.35	2+	3.66
42	9	Fe (oct)	6.65	1.35	2+	3.66
43	9	Fe (oct)	6.65	1.35	2+	3.66
44	9	Fe (oct)	6.65	1.35	2+	3.66
45	11	Fe (oct)	6.34	1.66	3+	4.14
46	11	Fe (oct)	6.35	1.65	3+	4.15
47	11	Fe (oct)	6.35	1.65	3+	4.15
48	11	Fe (oct)	6.34	1.66	3+	4.14
49	13	Fe (oct)	6.63	1.37	2+	3.65
50	13	Fe (oct)	6.63	1.37	2+	3.65
51	13	Fe (oct)	6.63	1.37	2+	3.65
52	13	Fe (oct)	6.63	1.37	2+	3.65
53	15	Fe (oct)	6.34	1.66	3+	4.16
54	15	Fe (oct)	6.36	1.64	3+	4.15
55	15	Fe (oct)	6.36	1.64	3+	4.15
56	15	Fe (oct)	6.34	1.66	3+	4.16
57	17	Fe (oct)	6.66	1.34	2+	3.64
58	17	Fe (oct)	6.66	1.34	2+	3.64
59	17	Fe (oct)	6.65	1.35	2+	3.64
60	17	Fe (oct)	6.65	1.35	2+	3.64
61	19	Fe (oct)	6.34	1.66	3+	4.15

62	19	Fe (oct)	6.34	1.66	3+	4.16
63	19	Fe (oct)	6.34	1.66	3+	4.16
64	19	Fe (oct)	6.34	1.66	3+	4.15
65	21	Fe (oct)	6.62	1.38	2+	3.65
66	21	Fe (oct)	6.62	1.38	2+	3.65
67	21	Fe (oct)	6.62	1.38	2+	3.65
68	21	Fe (oct)	6.62	1.38	2+	3.65
69	23	Fe (oct)	6.34	1.66	3+	4.14
70	23	Fe (oct)	6.34	1.66	3+	4.16
71	23	Fe (oct)	6.34	1.66	3+	4.16
72	23	Fe (oct)	6.34	1.66	3+	4.14
73	25	Fe (oct)	6.34	1.66	3+	4.18
74	25	Fe (oct)	6.34	1.66	3+	4.18
75	25	Fe (oct)	6.35	1.65	3+	4.18
76	25	Fe (oct)	6.35	1.65	3+	4.18
77	1	O	7.08	-1.08	2-	0.51
78	1	O	7.09	-1.09	2-	0.50
79	1	O	7.09	-1.09	2-	0.50
80	1	O	7.08	-1.08	2-	0.51
81	1	O	7.12	-1.12	2-	0.07
82	1	O	7.11	-1.11	2-	0.06
83	1	O	7.11	-1.11	2-	0.06
84	1	O	7.12	-1.12	2-	0.07
85	3	O	7.12	-1.12	2-	0.18
86	3	O	7.12	-1.12	2-	0.18
87	3	O	7.12	-1.12	2-	0.18
88	3	O	7.12	-1.12	2-	0.18
89	3	O	7.16	-1.16	2-	0.14
90	3	O	7.16	-1.16	2-	0.14
91	3	O	7.16	-1.16	2-	0.14
92	3	O	7.16	-1.16	2-	0.14
93	5	O	7.17	-1.17	2-	0.00
94	5	O	7.18	-1.18	2-	0.04
95	5	O	7.18	-1.18	2-	0.04
96	5	O	7.17	-1.17	2-	0.00
97	5	O	7.18	-1.18	2-	0.03
98	5	O	7.18	-1.18	2-	0.03
99	5	O	7.14	-1.14	2-	0.02
100	5	O	7.14	-1.14	2-	0.02
101	7	O	7.17	-1.17	2-	0.10
102	7	O	7.17	-1.17	2-	0.10
103	7	O	7.17	-1.17	2-	0.10
104	7	O	7.17	-1.17	2-	0.10
105	7	O	7.15	-1.15	2-	0.10
106	7	O	7.15	-1.15	2-	0.10
107	7	O	7.15	-1.15	2-	0.10
108	7	O	7.15	-1.15	2-	0.10
109	9	O	7.16	-1.16	2-	0.02
110	9	O	7.16	-1.16	2-	0.02
111	9	O	7.18	-1.18	2-	0.04
112	9	O	7.18	-1.18	2-	0.04
113	9	O	7.18	-1.18	2-	0.04
114	9	O	7.16	-1.16	2-	0.03

115	9	O	7.18	-1.18	2-	0.04
116	9	O	7.16	-1.16	2-	0.03
117	11	O	7.15	-1.15	2-	0.10
118	11	O	7.15	-1.15	2-	0.10
119	11	O	7.15	-1.15	2-	0.10
120	11	O	7.15	-1.15	2-	0.10
121	11	O	7.17	-1.17	2-	0.10
122	11	O	7.17	-1.17	2-	0.10
123	11	O	7.17	-1.17	2-	0.10
124	11	O	7.17	-1.17	2-	0.10
125	13	O	7.17	-1.17	2-	0.02
126	13	O	7.19	-1.19	2-	0.04
127	13	O	7.19	-1.19	2-	0.04
128	13	O	7.17	-1.17	2-	0.02
129	13	O	7.17	-1.17	2-	0.05
130	13	O	7.16	-1.16	2-	0.01
131	13	O	7.17	-1.17	2-	0.05
132	13	O	7.16	-1.16	2-	0.01
133	15	O	7.16	-1.16	2-	0.10
134	15	O	7.16	-1.16	2-	0.10
135	15	O	7.16	-1.16	2-	0.10
136	15	O	7.16	-1.16	2-	0.10
137	15	O	7.14	-1.14	2-	0.10
138	15	O	7.15	-1.15	2-	0.10
139	15	O	7.15	-1.15	2-	0.10
140	15	O	7.14	-1.14	2-	0.10
141	17	O	7.17	-1.17	2-	0.04
142	17	O	7.18	-1.18	2-	0.02
143	17	O	7.18	-1.18	2-	0.02
144	17	O	7.17	-1.17	2-	0.04
145	17	O	7.18	-1.18	2-	0.02
146	17	O	7.16	-1.16	2-	0.04
147	17	O	7.16	-1.16	2-	0.04
148	17	O	7.19	-1.19	2-	0.02
149	19	O	7.14	-1.14	2-	0.10
150	19	O	7.14	-1.14	2-	0.10
151	19	O	7.14	-1.14	2-	0.10
152	19	O	7.14	-1.14	2-	0.10
153	19	O	7.16	-1.16	2-	0.11
154	19	O	7.16	-1.16	2-	0.10
155	19	O	7.16	-1.16	2-	0.10
156	19	O	7.16	-1.16	2-	0.10
157	21	O	7.18	-1.18	2-	0.04
158	21	O	7.16	-1.16	2-	0.01
159	21	O	7.16	-1.16	2-	0.01
160	21	O	7.18	-1.18	2-	0.04
161	21	O	7.17	-1.17	2-	0.00
162	21	O	7.17	-1.17	2-	0.00
163	21	O	7.19	-1.19	2-	0.04
164	21	O	7.19	-1.19	2-	0.04
165	23	O	7.17	-1.17	2-	0.14
166	23	O	7.17	-1.17	2-	0.14
167	23	O	7.17	-1.17	2-	0.14

168	23	O	7.17	-1.17	2-	0.14
169	23	O	7.12	-1.12	2-	0.18
170	23	O	7.12	-1.12	2-	0.18
171	23	O	7.12	-1.12	2-	0.18
172	23	O	7.12	-1.12	2-	0.18
173	25	O	7.09	-1.09	2-	0.50
174	25	O	7.08	-1.08	2-	0.51
175	25	O	7.08	-1.08	2-	0.51
176	25	O	7.09	-1.09	2-	0.50
177	25	O	7.11	-1.11	2-	0.06
178	25	O	7.12	-1.12	2-	0.07
179	25	O	7.12	-1.12	2-	0.07
180	25	O	7.11	-1.11	2-	0.06

Table S5. Calculated magnetic moments, Bader charges and oxidation states derived from the charges from the DFT calculations for a tet$_1$ Fe_3O_4 (111) slab with 23 atomic layers and a 2×2 surface unit cell. Each line represents one atom in the calculated system. The data is presented in blocks of the tetrahedral irons Fe (tet), the octahedral irons Fe (oct), and the oxygens O. The first column provides a numeric label for each atom, the second numbers the atomic layer in which the specific atom is located, the third gives the element and configuration for Fe, the fourth the Bader charge, the fifth the relative atomic charge as the difference between the element specific number of valence electrons used in the DFT calculations (8 for Fe, 6 for O) and the Bader charge, the sixth the oxidation states derived from the relative atomic charge and seventh the magnetic moment. Charges are given in elementary charges (q) and magnetic moments in Bohr magnetons (μ_B)

magnetic moments in Bohr magnetons (μ_B)

#	Layer	Element	Electronic Bader charge (-q)	Relative atomic charge (q)	Oxidation State	Magnetic Moment (μ_B)
1	1	Fe (tet)	6.71	1.29	2+	-3.52
2	1	Fe (tet)	6.71	1.29	2+	-3.52
3	1	Fe (tet)	6.71	1.29	2+	-3.52
4	1	Fe (tet)	6.71	1.29	2+	-3.52
5	5	Fe (tet)	6.38	1.62	3+	-4.05
6	5	Fe (tet)	6.38	1.62	3+	-4.05
7	5	Fe (tet)	6.38	1.62	3+	-4.05
8	5	Fe (tet)	6.38	1.62	3+	-4.05
9	7	Fe (tet)	6.35	1.65	3+	-4.07
10	7	Fe (tet)	6.35	1.65	3+	-4.07
11	7	Fe (tet)	6.35	1.65	3+	-4.07
12	7	Fe (tet)	6.35	1.65	3+	-4.07

13	11	Fe (tet)	6.38	1.62	3+	-4.06
14	11	Fe (tet)	6.37	1.63	3+	-4.06
15	11	Fe (tet)	6.38	1.62	3+	-4.06
16	11	Fe (tet)	6.37	1.63	3+	-4.06
17	13	Fe (tet)	6.37	1.63	3+	-4.06
18	13	Fe (tet)	6.38	1.62	3+	-4.06
19	13	Fe (tet)	6.37	1.63	3+	-4.06
20	13	Fe (tet)	6.38	1.62	3+	-4.06
21	17	Fe (tet)	6.35	1.65	3+	-4.07
22	17	Fe (tet)	6.35	1.65	3+	-4.07
23	17	Fe (tet)	6.35	1.65	3+	-4.07
24	17	Fe (tet)	6.35	1.65	3+	-4.07
25	19	Fe (tet)	6.38	1.62	3+	-4.05
26	19	Fe (tet)	6.38	1.62	3+	-4.05
27	19	Fe (tet)	6.38	1.62	3+	-4.05
28	19	Fe (tet)	6.38	1.62	3+	-4.05
29	23	Fe (tet)	6.71	1.29	2+	-3.52
30	23	Fe (tet)	6.71	1.29	2+	-3.52
31	23	Fe (tet)	6.71	1.29	2+	-3.52
32	23	Fe (tet)	6.71	1.29	2+	-3.52
33	3	Fe (oct)	6.30	1.70	3+	4.15
34	3	Fe (oct)	6.30	1.70	3+	4.15
35	3	Fe (oct)	6.30	1.70	3+	4.15
36	3	Fe (oct)	6.30	1.70	3+	4.15
37	3	Fe (oct)	6.29	1.71	3+	4.15
38	3	Fe (oct)	6.30	1.70	3+	4.15
39	3	Fe (oct)	6.29	1.71	3+	4.15
40	3	Fe (oct)	6.30	1.70	3+	4.15
41	3	Fe (oct)	6.30	1.70	3+	4.15
42	3	Fe (oct)	6.29	1.71	3+	4.15
43	3	Fe (oct)	6.30	1.70	3+	4.15
44	3	Fe (oct)	6.29	1.71	3+	4.15
45	6	Fe (oct)	6.29	1.71	3+	4.15
46	6	Fe (oct)	6.28	1.72	3+	4.15
47	6	Fe (oct)	6.29	1.71	3+	4.15
48	6	Fe (oct)	6.28	1.72	3+	4.15
49	9	Fe (oct)	6.28	1.72	3+	4.16
50	9	Fe (oct)	6.63	1.37	2+	3.66
51	9	Fe (oct)	6.28	1.72	3+	4.16
52	9	Fe (oct)	6.63	1.37	2+	3.66
53	9	Fe (oct)	6.63	1.37	2+	3.66
54	9	Fe (oct)	6.27	1.73	3+	4.16
55	9	Fe (oct)	6.63	1.37	2+	3.66
56	9	Fe (oct)	6.27	1.73	3+	4.16
57	9	Fe (oct)	6.29	1.71	3+	4.13
58	9	Fe (oct)	6.29	1.71	3+	4.13
59	9	Fe (oct)	6.29	1.71	3+	4.13
60	9	Fe (oct)	6.29	1.71	3+	4.13
61	12	Fe (oct)	6.63	1.37	2+	3.64
62	12	Fe (oct)	6.63	1.37	2+	3.64
63	12	Fe (oct)	6.63	1.37	2+	3.64
64	12	Fe (oct)	6.63	1.37	2+	3.64
65	15	Fe (oct)	6.28	1.72	3+	4.16

66	15	Fe (oct)	6.63	1.37	2+	3.66
67	15	Fe (oct)	6.28	1.72	3+	4.16
68	15	Fe (oct)	6.63	1.37	2+	3.66
69	15	Fe (oct)	6.63	1.37	2+	3.66
70	15	Fe (oct)	6.27	1.73	3+	4.16
71	15	Fe (oct)	6.63	1.37	2+	3.66
72	15	Fe (oct)	6.27	1.73	3+	4.16
73	15	Fe (oct)	6.29	1.71	3+	4.13
74	15	Fe (oct)	6.29	1.71	3+	4.13
75	15	Fe (oct)	6.29	1.71	3+	4.13
76	15	Fe (oct)	6.29	1.71	3+	4.13
77	18	Fe (oct)	6.28	1.72	3+	4.15
78	18	Fe (oct)	6.29	1.71	3+	4.15
79	18	Fe (oct)	6.28	1.72	3+	4.15
80	18	Fe (oct)	6.29	1.71	3+	4.15
81	21	Fe (oct)	6.30	1.70	3+	4.15
82	21	Fe (oct)	6.29	1.71	3+	4.15
83	21	Fe (oct)	6.30	1.70	3+	4.15
84	21	Fe (oct)	6.29	1.71	3+	4.15
85	21	Fe (oct)	6.29	1.71	3+	4.15
86	21	Fe (oct)	6.30	1.70	3+	4.15
87	21	Fe (oct)	6.29	1.71	3+	4.15
88	21	Fe (oct)	6.30	1.70	3+	4.15
89	21	Fe (oct)	6.30	1.70	3+	4.15
90	21	Fe (oct)	6.30	1.70	3+	4.15
91	21	Fe (oct)	6.30	1.70	3+	4.15
92	21	Fe (oct)	6.30	1.70	3+	4.15
93	2	O	7.10	-1.10	2-	0.25
94	2	O	7.10	-1.10	2-	0.25
95	2	O	7.10	-1.10	2-	0.25
96	2	O	7.10	-1.10	2-	0.25
97	2	O	7.10	-1.10	2-	0.25
98	2	O	7.10	-1.10	2-	0.25
99	2	O	7.10	-1.10	2-	0.25
100	2	O	7.10	-1.10	2-	0.25
101	2	O	7.10	-1.10	2-	0.25
102	2	O	7.10	-1.10	2-	0.25
103	2	O	7.10	-1.10	2-	0.25
104	2	O	7.10	-1.10	2-	0.25
105	2	O	7.11	-1.11	2-	0.48
106	2	O	7.11	-1.11	2-	0.48
107	2	O	7.11	-1.11	2-	0.48
108	2	O	7.11	-1.11	2-	0.48
109	4	O	7.17	-1.17	2-	0.10
110	4	O	7.16	-1.16	2-	0.11
111	4	O	7.17	-1.17	2-	0.10
112	4	O	7.16	-1.16	2-	0.11
113	4	O	7.17	-1.17	2-	0.10
114	4	O	7.17	-1.17	2-	0.10
115	4	O	7.17	-1.17	2-	0.10
116	4	O	7.17	-1.17	2-	0.10
117	4	O	7.16	-1.16	2-	0.10
118	4	O	7.17	-1.17	2-	0.10

119	4	O	7.16	-1.16	2-	0.10
120	4	O	7.17	-1.17	2-	0.10
121	4	O	7.15	-1.15	2-	0.01
122	4	O	7.15	-1.15	2-	0.01
123	4	O	7.15	-1.15	2-	0.01
124	4	O	7.15	-1.15	2-	0.01
125	8	O	7.16	-1.16	2-	0.14
126	8	O	7.16	-1.16	2-	0.14
127	8	O	7.16	-1.16	2-	0.14
128	8	O	7.16	-1.16	2-	0.14
129	8	O	7.18	-1.18	2-	0.09
130	8	O	7.18	-1.18	2-	0.15
131	8	O	7.18	-1.18	2-	0.09
132	8	O	7.18	-1.18	2-	0.15
133	8	O	7.18	-1.18	2-	0.15
134	8	O	7.18	-1.18	2-	0.09
135	8	O	7.18	-1.18	2-	0.15
136	8	O	7.18	-1.18	2-	0.09
137	8	O	7.19	-1.19	2-	0.11
138	8	O	7.19	-1.19	2-	0.11
139	8	O	7.19	-1.19	2-	0.11
140	8	O	7.19	-1.19	2-	0.11
141	10	O	7.16	-1.16	2-	0.10
142	10	O	7.16	-1.16	2-	0.10
143	10	O	7.16	-1.16	2-	0.10
144	10	O	7.16	-1.16	2-	0.10
145	10	O	7.18	-1.18	2-	0.09
146	10	O	7.17	-1.17	2-	0.03
147	10	O	7.18	-1.18	2-	0.09
148	10	O	7.17	-1.17	2-	0.03
149	10	O	7.20	-1.20	2-	0.06
150	10	O	7.20	-1.20	2-	0.06
151	10	O	7.20	-1.20	2-	0.06
152	10	O	7.20	-1.20	2-	0.06
153	10	O	7.17	-1.17	2-	0.03
154	10	O	7.18	-1.18	2-	0.09
155	10	O	7.17	-1.17	2-	0.03
156	10	O	7.18	-1.18	2-	0.09
157	14	O	7.16	-1.16	2-	0.10
158	14	O	7.16	-1.16	2-	0.10
159	14	O	7.16	-1.16	2-	0.10
160	14	O	7.16	-1.16	2-	0.10
161	14	O	7.17	-1.17	2-	0.03
162	14	O	7.18	-1.18	2-	0.09
163	14	O	7.17	-1.17	2-	0.03
164	14	O	7.18	-1.18	2-	0.09
165	14	O	7.18	-1.18	2-	0.09
166	14	O	7.17	-1.17	2-	0.03
167	14	O	7.18	-1.18	2-	0.09
168	14	O	7.17	-1.17	2-	0.03
169	14	O	7.20	-1.20	2-	0.06
170	14	O	7.20	-1.20	2-	0.06
171	14	O	7.20	-1.20	2-	0.06

172	14	O	7.20	-1.20	2-	0.06
173	16	O	7.16	-1.16	2-	0.14
174	16	O	7.16	-1.16	2-	0.14
175	16	O	7.16	-1.16	2-	0.14
176	16	O	7.16	-1.16	2-	0.14
177	16	O	7.18	-1.18	2-	0.15
178	16	O	7.18	-1.18	2-	0.09
179	16	O	7.18	-1.18	2-	0.15
180	16	O	7.18	-1.18	2-	0.09
181	16	O	7.19	-1.19	2-	0.11
182	16	O	7.19	-1.19	2-	0.11
183	16	O	7.19	-1.19	2-	0.11
184	16	O	7.19	-1.19	2-	0.11
185	16	O	7.18	-1.18	2-	0.09
186	16	O	7.18	-1.18	2-	0.15
187	16	O	7.18	-1.18	2-	0.09
188	16	O	7.18	-1.18	2-	0.15
189	20	O	7.17	-1.17	2-	0.10
190	20	O	7.17	-1.17	2-	0.10
191	20	O	7.17	-1.17	2-	0.10
192	20	O	7.17	-1.17	2-	0.10
193	20	O	7.17	-1.17	2-	0.10
194	20	O	7.17	-1.17	2-	0.11
195	20	O	7.17	-1.17	2-	0.10
196	20	O	7.16	-1.16	2-	0.11
197	20	O	7.16	-1.16	2-	0.10
198	20	O	7.17	-1.17	2-	0.10
199	20	O	7.16	-1.16	2-	0.10
200	20	O	7.17	-1.17	2-	0.10
201	20	O	7.15	-1.15	2-	0.01
202	20	O	7.15	-1.15	2-	0.01
203	20	O	7.15	-1.15	2-	0.01
204	20	O	7.15	-1.15	2-	0.01
205	22	O	7.10	-1.10	2-	0.25
206	22	O	7.10	-1.10	2-	0.25
207	22	O	7.10	-1.10	2-	0.25
208	22	O	7.10	-1.10	2-	0.25
209	22	O	7.10	-1.10	2-	0.25
210	22	O	7.10	-1.10	2-	0.25
211	22	O	7.10	-1.10	2-	0.25
212	22	O	7.10	-1.10	2-	0.25
213	22	O	7.10	-1.10	2-	0.25
214	22	O	7.10	-1.10	2-	0.25
215	22	O	7.10	-1.10	2-	0.25
216	22	O	7.10	-1.10	2-	0.25
217	22	O	7.11	-1.11	2-	0.48
218	22	O	7.11	-1.11	2-	0.48
219	22	O	7.11	-1.11	2-	0.48
220	22	O	7.11	-1.11	2-	0.48

Wulff shapes of unsupported magnetite nanoparticles

In Santos-Carballal et al.[26], the surface energies for (001) and (111) were reported to be 0.96 J/m^2 and 1.09 J/m^2, respectively. In that publication, a cubic shape with dominating (001) facets is presented for unsupported magnetite nanoparticles. Only the corners are truncated with (111) facets. The facet fractions of the {001} and {111} surfaces are about 60% and 40%, respectively. A Wulff shape using that data is shown in Figure S11a.

The shapes of unsupported nanoparticles were also predicted from the surface energies in Table 3. The predicted facet fractions are presented in Table S6. Assuming {001} distorted bulk truncated (DBT) and {111} iron tetrahedral (Fe-tet$_1$) terminations, octahedral-shaped nanoparticles were obtained with dominant {111} facets. The {111} dominance decreases with temperature from a facet fraction of 95% at 0 K to 63% at 800 K (See Figures S11b and S12). Alternatively, assuming a subsurface cation vacancy (SCV) termination for the {001} surfaces resulted in cubic nanoparticles dominated by {001} facets. At low temperatures, only {001} facets were predicted, whereas at 600 K and 800 K, {111} facets were emerging with facet fractions of 22% and 56%, respectively (See Figure S13). Depending on the growth conditions, cubic nanoparticles or even complex shapes such as stars can be synthesized; however, octahedrally shaped particles are often observed[27-29]. Using the data from Ref.[30,31] for {001} DBT and {111} tet$_1$ gives similar results to our data.

a b

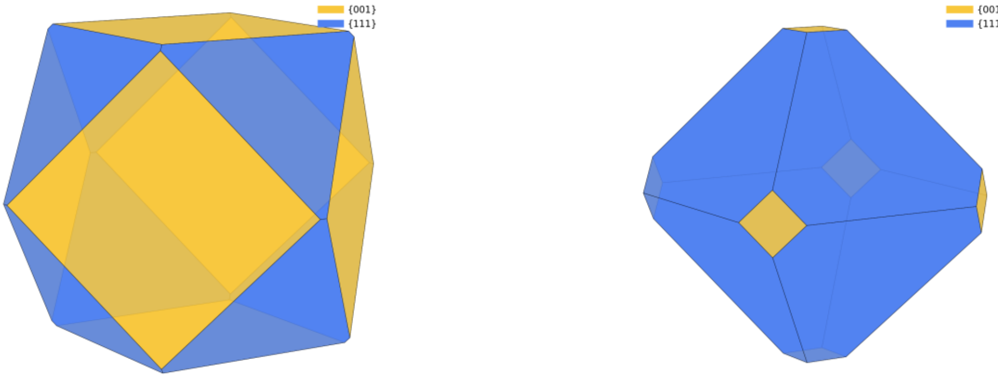

Figure S11. Wulff constructions for unsupported magnetite nanoparticles at 0 K, a) based on data from Santos-Carballal et al.[26] and b) based on surface energies from this study for {001} DBT and {111} tet$_1$. {001} and {111} surfaces are colored in yellow and blue, respectively.

a b

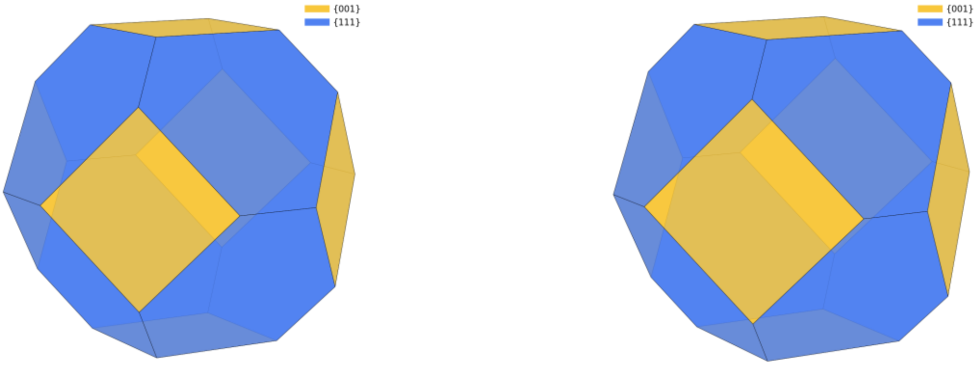

Figure S12. Wulff constructions for unsupported magnetite nanoparticles based on surface free energies from this study for {001} DBT and {111} tet$_1$ at a) 600 K and b) 800 K. {001} and {111} surfaces are colored in yellow and blue, respectively.

a b

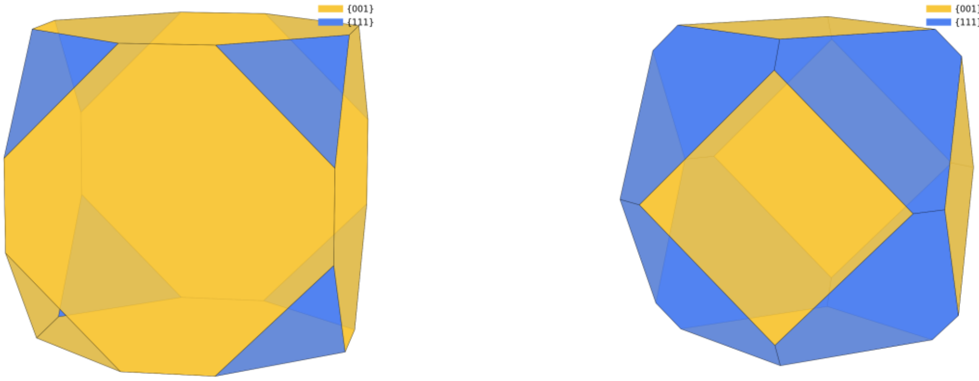

Figure S13. Wulff constructions for unsupported magnetite nanoparticles based on surface free energies from this study for {001} SCV and {111} tet$_1$ at a) 600 K and b) 800 K. {001} and {111} surfaces are colored in yellow and blue, respectively.

Wulff shapes of supported magnetite nanoparticles

In Fig. S14, the Winterbottom constructions for supported magnetite nanoparticles from Gibbs free surface energies from this study for {001} SCV and {111} tet_1 surfaces are presented for different temperatures. The effective surface free energy is smaller than minus one times the smallest Fe_3O_4 surface energy up to 450 K. This corresponds to complete wetting and would not produce well-defined NP at the substrate. This is in contradiction to the experiments. From 500 K, partial wetting is expected. The {001} facet fractions are 49 and 30 %, for 600 and 800 K, respectively. The {111} facet fractions are 12 and 31 %, for 600 and 800 K, respectively. The missing NP surface areas to reach 100 % are the interface areas towards the substrate.

a b

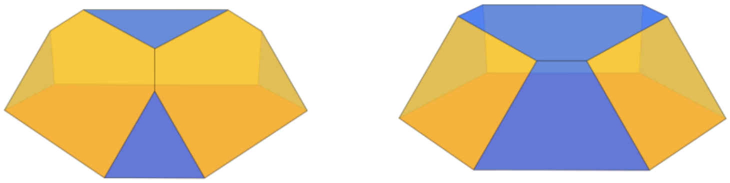

Figure S14. Winterbottom constructions for supported magnetite nanoparticles from Gibbs free surface energies from this study for {001} SCV and {111} tet_1 colored in yellow and blue, respectively, for an oxygen pressure of $p = 8 \cdot 10^{-7}$ mbar and at a) 600 K and b) 800 K.

Table S6. Predicted facet fractions of {001}, {111}, and interface facets for supported NP using the Winterbottom construction (See Methods). Fractions are given in percent (rounded to the first digit) of the total NP surface area, including the interface area for NPs showing {111} tet_1 and either {001} DBT or {001} SCV facets. Predictions based on the terminations and energies from Ref.[26] are also presented

Surface terminations	Temperature [K]	Facet fraction [%]		
		**{001}**	**{111}**	**interface**
{001} DBT & {111} tet_1	0	4.0	64.0	32.1
	450	21.8	40.0	38.2
	600	24.1	37.5	38.4
	800	26.5	34.9	38.6
{001} SCV & {111} tet_1	450	Complete wetting		
	600	49.2	12.1	38.7
	800	29.8	31.4	38.8
Santos-Carballal et al.[26]	0 - 800	38.5	22.6	38.9

Supplementary References

1. Schindelin J, *et al.* Fiji: an open-source platform for biological-image analysis. *Nature Methods* **9**, 676-682 (2012).

2. Kim W, *et al.* A new method for the identification and quantification of magnetite–maghemite mixture using conventional X-ray diffraction technique. *Talanta* **94**, 348-352 (2012).

3. Arndt B, *et al.* Carboxylic acid induced near-surface restructuring of a magnetite surface. *Communications Chemistry* **2**, 92 (2019).

4. Creutzburg M, *et al.* Heterogeneous adsorption and local ordering of formate on a magnetite surface. *The Journal of Physical Chemistry Letters* **12**, 3847-3852 (2021).

5. Gürsoy E, Vonbun-Feldbauer GB, Meißner RH. Oxidation-state dynamics and emerging patterns in magnetite. *The Journal of Physical Chemistry Letters* **14**, 6800-6807 (2023).

6. Meng Y, *et al.* Prediction on morphologies and phase equilibrium diagram of iron oxides nanoparticles. *Applied Surface Science* **480**, 478-486 (2019).

7. Kresse G, Furthmüller J. Efficiency of ab-initio total energy calculations for metals and semiconductors using a plane-wave basis set. *Computational Materials Science* **6**, 15-50 (1996).

8. Kresse G, Furthmüller J. Efficient iterative schemes for ab initio total-energy calculations using a plane-wave basis set. *Physical Review B* **54**, 11169 (1996).

9. Kresse G, Hafner J. Ab initio molecular dynamics for liquid metals. *Physical Review B* **47**, 558 (1993).

10. Kresse G, Hafner J. Ab initio molecular-dynamics simulation of the liquid-metal–amorphous-semiconductor transition in germanium. *Physical Review B* **49**, 14251 (1994).

11. Perdew JP, Burke K, Ernzerhof M. Generalized gradient approximation made simple. *Physical Review Letters* **77**, 3865 (1996).

12. Dudarev SL, Botton GA, Savrasov SY, Humphreys C, Sutton AP. Electron-energy-loss spectra and the structural stability of nickel oxide: An LSDA+ U study. *Physical Review B* **57**, 1505 (1998).

13. Blöchl PE. Projector augmented-wave method. *Physical Review B* **50**, 17953 (1994).

14. Sellschopp K. Understanding interfaces in metal-oxide/organic-acid hybrid materials from first-principles calculations. Doctoral Dissertation (2021).

15. Blöchl PE, Jepsen O, Andersen OK. Improved tetrahedron method for Brillouin-zone integrations. *Physical Review B* **49**, 16223 (1994).

16. Neugebauer J, Scheffler M. Adsorbate-substrate and adsorbate-adsorbate interactions of Na and K adlayers on Al (111). *Physical Review B* **46**, 16067 (1992).

17. Bader RF. Atoms in molecules. *Accounts of Chemical Research* **18**, 9-15 (1985).

18. Henkelman G, Arnaldsson A, Jónsson H. A fast and robust algorithm for Bader decomposition of charge density. *Computational Materials Science* **36**, 354-360 (2006).

19. Tang W, Sanville E, Henkelman G. A grid-based Bader analysis algorithm without lattice bias. *Journal of Physics: Condensed Matter* **21**, 084204 (2009).

20. Sanville E, Kenny SD, Smith R, Henkelman G. Improved grid-based algorithm for Bader charge allocation. *Journal of Computational Chemistry* **28**, 899-908 (2007).

21. Liu H, Di Valentin C. Band gap in magnetite above Verwey temperature induced by symmetry breaking. *The Journal of Physical Chemistry C* **121**, 25736-25742 (2017).

22. Gürsoy E, Meißner RH, Vonbun-Feldbauer GB. Atomistic modeling of functionalized magnetite surfaces with oxidation states. *The Journal of Physical Chemistry Letters* **16**, 6765-6770 (2025).

23. Reuter K, Scheffler M. Composition, structure, and stability of RuO_2 (110) as a function of oxygen pressure. *Physical Review B* **65**, 035406 (2001).

24. Chase MW. NIST-JANAF thermochemical tables. *Journal of Physical and Chemical Reference Data* **28**, 1951 (1998).

25. Sargeant E, Illas F, Rodriguez P, Calle-Vallejo F. Importance of the gas-phase error correction for O_2 when using DFT to model the oxygen reduction and evolution reactions. *Journal of Electroanalytical Chemistry* **896**, 115178 (2021).

26. Santos-Carballal D, Roldan A, Grau-Crespo R, de Leeuw NH. A DFT study of the structures, stabilities and redox behaviour of the major surfaces of magnetite Fe_3O_4. *Physical Chemistry Chemical Physics* **16**, 21082-21097 (2014).

27. Cornell RM, Schwertmann U. *The iron oxides: structure, properties, reactions, occurrences, and uses*. Wiley-vch Weinheim (2003).

28. Zhao L, *et al.* Morphology-controlled synthesis of magnetites with nanoporous structures and excellent magnetic properties. *Chemistry of Materials* **20**, 198-204 (2008).

29. Feld A, *et al.* Chemistry of shape-controlled iron oxide nanocrystal formation. *ACS Nano* **13**, 152-162 (2018).

30. Kraushofer F, *et al.* Oxygen-terminated (1×1) reconstruction of reduced magnetite Fe_3O_4 (111). *The Journal of Physical Chemistry Letters* **14**, 3258-3265 (2023).

31. Sombut P, *et al.* The surface phase diagram of Fe_3O_4 (001) revisited. *RSC Applied Interfaces* **2**, 673–683 (2025).
