# Supplementary material for: Equilibrium shape and surface termination of supported magnetite nanoparticles
Source: Commun Chem. 2026 Apr 11;9:158. doi: 10.1038/s42004-026-02008-4 (PMC13083894; doi:10.1038/s42004-026-02008-4)
Supplement: Supplementary file 2 — Description of Additional Supplementary Files [file 42004_2026_2008_MOESM2_ESM.pdf]

Description of Additional Supplementary Files:

File: Supplementary Data 1

Description: 001_DBT

File: Supplementary Data 2

Description: 111_tet1
